# Selecting the Best Animal Model of Parkinson’s Disease for Your Research Purpose: Insight from *in vivo* PET Imaging Studies

**DOI:** 10.2174/1570159X21666230216101659

**Published:** 2023-04-12

**Authors:** Caroline Cristiano Real, Karina Henrique Binda, Majken Borup Thomsen, Thea Pinholt Lillethorup, David James Brooks, Anne Marlene Landau

**Affiliations:** 1 Department of Nuclear Medicine and PET Center, Aarhus University Hospital, Aarhus, Denmark;; 2 Translational Neuropsychiatry Unit, Department of Clinical Medicine, Aarhus University, Aarhus, Denmark;; 3 Institute of Translational and Clinical Research, University of Newcastle, Upon Tyne, UK

**Keywords:** Animal models, Parkinson’s disease, rodent, non-human primate, minipig, alpha-synuclein, positron emission tomography, autoradiography

## Abstract

Parkinson’s disease (PD) is a debilitating neurodegenerative multisystem disorder leading to motor and non-motor symptoms in millions of individuals. Despite intense research, there is still no cure, and early disease biomarkers are lacking. Animal models of PD have been inspired by basic elements of its pathogenesis, such as dopamine dysfunction, alpha-synuclein accumulation, neuroinflammation and disruption of protein degradation, and these have been crucial for a deeper understanding of the mechanisms of pathology, the identification of biomarkers, and evaluation of novel therapies. Imaging biomarkers are non-invasive tools to assess disease progression and response to therapies; their discovery and validation have been an active field of translational research. Here, we highlight different considerations of animal models of PD that can be applied to future research, in terms of their suitability to answer different research questions. We provide the reader with important considerations of the best choice of model to use based on the disease features of each model, including issues related to different species. In addition, positron emission tomography studies conducted in PD animal models in the last 5 years are presented. With a variety of different species, interventions and genetic information, the choice of the most appropriate model to answer research questions can be daunting, especially since no single model recapitulates all aspects of this complex disorder. Appropriate animal models in conjunction with *in vivo* molecular imaging tools, if selected properly, can be a powerful combination for the assessment of novel therapies and developing tools for early diagnosis.

## 1 INTRODUCTION

Parkinson’s disease (PD) is an age-related progressive neurodegenerative disorder characterized by neuronal and neuritic accumulation of misfolded and aggregated alpha-synuclein (α-syn) containing inclusions called Lewy bodies and Lewy neurites in brain regions targeting the substantia nigra *pars compacta* (SNpc) and also peripheral organs. Loss of dopaminergic neurons in the SNpc causes a reduction in available dopamine (DA) in the striatum, which affects signalling to the motor cortex and initiates the most characteristic clinical symptoms of PD, namely limb bradykinesia in combination with rigidity and/or rest tremor [1, 2]. The spread of α-syn inclusions and neurodegeneration affect not only the dopaminergic system but also the serotonergic, noradrenergic and cholinergic systems. These are responsible for the non-motor features of PD, including sleep, autonomic and psychiatric dysfunctions, such as insomnia, rapid eye movement (REM), sleep behaviour disorder (RBD), constipation, depression, and hyposmia [2]. The development of the non-motor features can occur both prodromally and at a later stage of PD. Failure to clear α-syn or its overexpression, misfolding, aggregation and propagation is the underlying mechanism driving Lewy body disorders; this molecular pathogenesis also leads to altered mitochondrial function, oxidative stress, and neuroinflammation/glial activation. While age is the greatest risk factor for PD, multiple aspects have been suggested to play a role in the aetiology of PD, including inflammation, gut microbiome composition [3, 4], and exposure to environmental factors, including infection, pesticides and air pollution [5, 6]. The majority of PD cases are idiopathic though 5-40% have a genetic origin depending on ethnicity. Towards the end of the 20^th^ century, a possible link between glucocerebrosidase gene (*GBA*) mutations and PD was highlighted. Approximately 5-15% of PD patients have mutations in the *GBA* gene, which encodes the lysosomal enzyme glucocerebrosidase A (GCase), making it the highest genetic risk factor for PD. In Caucasians, GBA mutations are more prevalent than other gene mutations associated with familial PD, including leucine-rich repeat kinase 2 gene (LRRK2), α-syn (SNCA), and PARK2 (also known as PRKN/Parkin) [7].

The prodromal phase of PD, where α-syn spreads and neurodegeneration is initiated, can last 10-20 years prior to the classical clinical motor phase of PD [8]. The heterogeneity in the aetiology of the disease reflects the marked heterogeneity in the clinical phenotype, and defining meaningful phenotypes of PD is necessary for targeted therapy [9]. The genetic and environmental factors that increase the risk of PD can lead to subtypes of prodromal PD, including RBD, and have led to the concept of brain-first or body-first PD [10-12]. DA replacement therapy with levodopa is the most effective symptomatic treatment for motor symptoms of PD, but a major treatment challenge is its side effects that develop over time, including fluctuating motor responses, dyskinesias and compulsive behaviours. In the early phase of PD, DA agonists, monoamine oxidase B (MAO-B) and anticholinergic agents can be used for treatment and later catechol-*O*-methyltransferase inhibitors can be combined with levodopa to increase its half-life in order to delay its adverse effects, but in the long term, they no longer have sustained efficacy [13]. Evidence from randomised controlled trials over the past 5 years has confirmed that amantadine can be used to suppress levodopainduced dyskinesias in patients with PD, and clinical studies have also provided support for its potential to reduce motor fluctuations [14]; however, additional studies are necessary. Surgical treatments can be an option for patients who have severe levodopa-associated side effects. Most common is subthalamic nuclei (STN) deep brain stimulation (DBS), which usually provides prolonged and efficient control of motor symptoms and a reduction in dopaminergic medication, but can worsen cognitive deficits and has variable effects on other symptoms that require dedicated management and personalized care for each patient. Early STN DBS in PD reduces long-term medication costs [15]. Ablative procedures with focused ultrasound can now be performed unilaterally to reduce tremors. However, there is still no standard long-term treatment that is beneficial for chronic PD patients [16].

There is evidence that modern lifestyles can influence the risk of developing neurodegenerative diseases, including PD. The adoption of positive lifestyle behaviours, like a healthy diet, proper sleep and physical activity routine, and social and cognitive engagement have been indicated as potential strategies to decrease the risk of developing PD and can lead to better management of the disease [17, 18]. Preclinical studies have demonstrated the importance of physical activity and dietary supplementation for slowing PD progression and reducing PD-associated pathology, including synaptic deficits and neuroinflammation [19-23]. Clinical trials are in progress to reinforce the importance of a healthy lifestyle in conjunction with PD patient treatment [24-26]. Aerobic exercise can be implemented immediately as a low-cost and easily accessible co-treatment for PD.

Despite extensive research efforts, there are still no effective tools to prevent PD onset, detect the disease in its early stages, or accurately predict the risk of disease progression. The lack of a precision medicine approach and a specific biomarker for early diagnosis may be the reason for the many failed clinical trials of novel protective drugs for PD. The limited treatment options for PD highlight the need for experimental animal models to test treatments and to improve our understanding of this complicated disease. In addition, it is important to use a proper non-invasive tool to help in early diagnosis, personalized medicine, and to follow the disease progression and treatment efficacy, such as positron emission tomography (PET), a unique tool for non-invasive *in vivo* molecular imaging. PET can detect objective alterations in the neurochemical machinery of the brain and help understand disease mechanisms, detect subclinical disease and diagnose PD in conjunction with the use of specific imaging biomarkers, termed radioligands. PET allows disease progression to be objectively monitored, making it a useful tool for assessing the validity of novel therapies [27, 28]. In this review, we present animal models of PD that are currently available, discussing their strengths and limitations in terms of their suitability to answer different research questions. Furthermore, since the use of appropriate animal models with PET imaging is a robust combination, we present combined studies from the last 5 years. Finally, we touch upon the relevant PET research for the development of novel PET ligands for the clinic and the use of animal models of PD to investigate the validity of these new markers.

## 2 PARKINSON’S DISEASE ANIMAL MODELS

A diverse range of models are available to test hypotheses and study specific aspects of PD pathology. In most cases, the development of animal models has been inspired by basic elements of PD pathogenesis, such as loss of dopaminergic neurons, α-syn accumulation, neuroinflammation, mitochondrial dysfunction, oxidative stress, and disruption of protein degradation. However, it is not feasible to replicate all aspects of the human disease completely since PD is a complex multisystem disorder [29]. Nevertheless, studies using PD animal models are frequently performed and have been found valuable for the development of symptomatic treatments, despite the known limitations regarding the complexity of the human disease. It is important to choose the most appropriate model carefully to best suit the main purpose of the study, such as investigating molecular mechanisms of behavioural symptoms, the response of symptoms to different therapeutics, whether drugs are neuroprotective, and trialling novel biomarkers. The ideal model must be experimentally rational and cost-effective. PD animal models can differ based on the type of animal species, the injected substance and injection area, the administered dose and the dosing paradigm (*e.g*., acute *vs*. chronic treatments). The choice of the successful model depends on a balance between the main scientific question and the strengths and limitations of the model. It is important to design the experiment in a way that minimizes the limitations of the model and increases the translational validity. It is also crucial to ask how pertinent is the question and how applicable are the methods utilized to answer them. The validation of animal models of PD is extremely important and often relies on behavioural assessments for each type of model. Examples of behavioural studies include the use of the cylinder test to detect asymmetry in a unilateral model, open field studies of locomotion, novel object recognition for the testing of cognition and memory, nociceptive threshold, and rotarod and gait assessments of motor coordination. In addition to behavioural tests, *postmortem* analysis must be used to confirm the pathology present in the model. The classical marker of dopaminergic cell degeneration is the loss of tyrosine hydroxylase (TH), a rate-limiting enzyme for DA synthesis, which is often measured using immunohistochemistry. To detect neuroinflammation, microglial activation is usually measured with the ionized calcium-binding adapter molecule 1 (Iba-1) antibody.

### 2.1 Animal Models: Choice of Species

Due to their potential and short life cycle, there is a wide availability of transgenic rodent models [30]. This makes them very useful for the initial testing of a new hypothesis as preliminary results can often be obtained rapidly. Studies using PD models indicate that aged animals are more susceptible to various model induction substances [31, 32], and importantly, perhaps less responsive to therapies [33]. Despite the short life span of rodents and PD being a disorder of primarily aged individuals slowly progressing over decades, the research community does not appear to base their study design on more appropriately aged animals, choosing instead to work with young adult animals. The use of aged animals can be a disadvantage due to increased risk of mortality and time limitations for studying progression of disease and long-term effects of treatments, and the costs associated with the extended maintenance of the animals at animal facilities [34]. This reproducibility challenge can be a contributing factor to the lack of predictive validity of neuroprotective efficacy of agents trialled in animals [35]. Furthermore, rodent neuroanatomy is not as complex as that of humans, which makes translational studies difficult. As an example, the rodent striatum appears as a single mass pierced by cortical fibers [36]. On the other hand, the non-human primate (NHP) model, which has a distinct caudate and putamen, has been valuable since the 1980s, when ground-breaking neuroanatomical and electrophysiological studies were performed, which enabled researchers to identify the distinct cortical-basal ganglia circuits responsible for the cardinal motor features of PD and study their pathophysiology [37, 38]. The NHP has the advantage of closely resembling human neuroanatomical complexity and has the motor and cognitive skills similar to those of humans, providing insight into clinical issues [39] and has much to offer in the search for PD modifying therapies [37]. The limitation of using NHPs as model species arises from ethical, practical, regulatory and financial considerations, which can largely hamper animal neuroscience [40].

Although rodents are often the species of choice for practical reasons, while the NHP has higher translational value, the use of porcine models has become increasingly common in neuroscience research [41, 42], specifically in PD. Their large brain has far greater complexity compared to rodents, which provides a more direct translation of human brain function in terms of health and disease [40, 43]. They share some anatomical brain similarities with humans, for example, the gyrification of the cortex and the neuroanatomy of the striatum. The striatum in pigs, unlike rodents, is divided by the internal capsule into two regions: caudate and putamen [43]. This division is similar to what is found in humans and can, therefore, directly contribute to the improvement of translational studies. Additionally, the porcine immune system is over 80% similar to that of human, while the overlap between rodents and human is only about 10% [44, 45]. Pigs have, for example, tonsils, their skin is very similar to human skin [46], and they are monogastric and omnivorous species, which make them an excellent model for studying intestinal immunology [47]. Domestic farm pigs are much larger (>300 kg) compared to other animals used in research (*e.g*., mice, rats, rabbits, macaque, vervet), which requires more space, higher costs, and is associated with greater difficulties in their handling [48]. The development of minipigs (*e.g*., the Göttingen minipig) specifically bred for research has made it possible to perform longitudinal studies in pigs due to their low adult body weight, slow growth rate, and a large brain. Minipigs are therefore more desirable for use in research because their size is not an issue using PET or MRI imaging modalities; thus, a longitudinal imaging study design can be employed, and their weight is also not a limiting factor in the cost of drug administration per kg.

In the last decade, a variety of minipig PD models have been introduced, all with their own strengths and limitations, including lesions with 6-hydroxydopamine (6-OHDA) [49] and 1-methyl-4-phenyl-1,2,3,6-tetrahydropyridine (MPTP) using different routes of administration [49-51], striatal inoculations with recombinant adeno-associated virus (AAV) vectors containing human α-syn mutations [52], and acute and chronic proteasome inhibition [53, 54]. Ongoing work aims to generate a genetic model of PD based on the overexpression of porcine α-syn [55]. PD-relevant treatments have been trialled, such as stem-cell transplantation [56-58] and STN-DBS [59]. The use of minipigs enables longitudinal and detailed *in vivo* imaging studies through conventional clinical brain imaging equipment with multiple PET tracers [56, 60-62]. The size of minipigs allows experiments with human medical devices [61, 63, 64], increasing the translation of the model to the human condition.

In addition to the commonly used models described above, alternative non-mammalian models have also been applied to PD research, such as zebrafish [65], goldfish [66], and drosophila [67]. More recently, 3-dimensional *in vitro* organoid models have gained popularity, and their use was recently reviewed along with other cell-based *in vitro* models [68].

To help the researchers from the PD field decide which model best fits their study hypothesis and which gaps still need to be filled for our improved understanding of the disease, the main mechanisms involved in the most frequently used mammalian PD models in recent years will be described below. In addition, we list which PET tracers were used with each model to evaluate potential therapeutic interventions in the last 5 years. Section 4 will be divided into the following models: (1) toxin-induced models: 6-OHDA, MPTP, rotenone, lipopolysaccharides (LPS), proteasome inhibition; (2) transgenic models; (3) α-syn models: AAV and preformed fibrils (PFFs); (4) Gut-first animal models. Reserpine and haloperidol models will not be discussed here since those models are associated with spontaneous recovery, have limitations for long-term use, and fail to show pathological characteristics [69]. Table **1** summarizes the main characteristics of each model listed in this review. Fig. (**1**) summarizes which models are available for each species.

## 3 POSITRON EMISSION TOMOGRAPHY RADIOLIGAND IMAGING

In PD, imaging of the dopaminergic system with radioligands binding to DA receptors and transporters is important for supporting diagnosis and studying disease progression and response to therapies [28, 70]. Dopaminergic imaging is commonly performed using PET or single-photon emission computerized tomography (SPECT). Other PET radioligands targeting the serotonin, noradrenaline and acetylcholine systems have been used to image molecular changes associated with non-motor aspects of PD [71-73]. PET radioligands of microglial activation [74], synaptic vesicle glycoprotein 2A (SV2A) density [75-78] and mitochondrial dysfunction [78] have also been used to study PD patients. Neuroimaging is also being used to understand the link between GBA gene mutations and the risk of developing PD and the severity of the disease, as recently reviewed [79]. PET data can be correlated with clinical and behavioural scores and fluid biomarkers in PD patients, providing more robustness to the diagnosis and evaluation, but the labelling and use of the most appropriate compounds are still a challenge. New horizons may be reached by developing tracers that specifically bind to proteins known to lead to the development or acceleration of PD, like α‐syn and proteins promoting neuroinflammation and synaptic dysfunction [27].

Neuroinflammation/glial activation may not only be a trigger for disease onset but may also promote the progression of PD. Several tracers have been developed as markers of microglial activation focusing on the hyperexpression of the 18 kDa translocator protein (TSPO). The first-generation tracer, which is still commonly used, is [^11^C]PK11195. Compared to first generation ligands, second generation ligands have improved signal-to-noise ratio and lower non-specific binding, but their binding is influenced by the TSPO polymorphism expressed [74], and so human subjects have to be genotyped to determine their ligand binding status. A new marker of neuroinflammation is the microglial expression of colony-stimulating factor 1 receptor **(**CSF1R), and radioligands for this target, including [^11^C]CPPC and [^11^C]GW2580, have been developed [80, 81], but they suffer from low signal-to-noise ratios. The search for improved CSF1R ligands is currently an active field of PET radioligand development research.

α-syn is a presynaptic protein involved in synaptic vesicle recycling and its abnormal aggregation is linked to PD. The development of a radioligand selectively targeting α-syn aggregates is a major unmet need in the PD field and would have utility for the early and differential diagnosis of PD and other synucleinopathies. Indeed, several research teams have undertaken this task and have labelled different α-syn binding ligands with radioactive labels [82, 83], including phenothiazines, indolinonedienes and chalcone-like derivatives, benzoxazoles, diaryl pyrazoles, and bisquinolines [74, 84]. These were prepared with the ambitious overarching aim of understanding potential correlations between α-syn load and distribution, clinical symptom severity and disease progression. The availability of such a ligand would allow the *in vivo* monitoring of the effects of new therapeutic strategies designed to inhibit α-syn aggregation and deposition and could play an important role in providing effective therapies to patients. Unfortunately, these trials have had limited success in developing a selective ligand for imaging α-syn aggregates in PD patients as both α-syn and amyloid fibrils have beta-sheeted structures to which these ligands bind. Additionally, the intracellular location of α-syn and the presence of higher concentrations of other protein aggregates, such as beta-amyloid plaques and tau in PD, can be problematic. Often more specific α-syn ligands, such as peptides, do not have adequate lipophilicity to passively cross the blood-brain barrier (BBB). In future attempts, labelled lead candidates will be trialled using *in vitro* and *in vivo* models, and the choice of the optimal animal model will be of critical importance.

Animal models are advantageous as they can be imaged longitudinally, at baseline, after the induction of a PD model and after a therapeutic intervention. This within-animal approach reduces variability when comparing *in vivo* functional data with *postmortem* histological measures of α-syn aggregation, neuroinflammation or DA decrease, when trialling novel experimental therapies not yet approved for human. Animal models can be scanned multiple times (with different radioligands, as needed) without the ethical issue of radiation dosimetry limits associated with human studies. Fig. (**2**) illustrates the main tracers used in preclinical PD studies.

In addition to PET, autoradiography can be used to corroborate *in vivo* PET data, and can be performed *in vitro* to validate a PET tracer before *in vivo* imaging studies are planned. Autoradiography can be used to visualise and quantify *in vitro* densities of specific target proteins. Since autoradiography requires *postmortem* fresh frozen tissue, it is performed only at a single timepoint. However, it has the advantage of avoiding confounding effects of tracer metabolism, blood flow, passage through the BBB and plasma protein binding, which can complicate PET measurements.

Parameters, such as binding potential and dissociation constant, can be quantified using autoradiography, which can be useful in the characterization of new potential PET ligands. Other molecular assays can also be relevant tools to support PET data, including immunohistochemistry and electron microscopy [85].

## 4 TYPES OF PARKINSON’S DISEASE ANIMAL MODELS

Fig. (**3**) summarizes the main primary pathophysiological mechanisms of the most common animal models of PD that will be described in the next sections.

### 4.1 Toxin-induced Models

The two most common “classical” PD models involve the use of neurotoxins, 6-OHDA and MPTP. These have been primarily employed for the testing of symptomatic but also putative neuroprotective therapies. In this context, there is a need for animals with clear and stable behavioural symptoms without spontaneous recovery. With the development of new therapies and new knowledge regarding PD, limitations of these acute toxin models have been pointed out, including the lack of protein aggregation/Lewy body-like pathology in the 6-OHDA model. The presence of Lewy body-like inclusions in monkey MPTP models has been reported, especially in aged animals [86-88]. In mice, there is an upregulation of α-syn and hyperphosphorylation of tau [89] without evidence of aggregation after MPTP [90]. An important aspect of the classic neurotoxin models is that they selectively and rapidly destroy catecholaminergic neurons, whereas in humans, the PD pathogenesis follows a progressive course over decades.

#### 4.1.1 6-Hydroxydopamine (6-OHDA)

6-OHDA, the first neurotoxin model for PD, has a similar structure to DA, and it is transported into dopaminergic neurons by the DA transporter (DAT). After injection, it leads to oxidative stress through the inhibition of mitochondrial complex I and the production of reactive oxygen species (ROS) [91]. To induce the model, it is necessary to perform sterile stereotaxic surgery since 6-OHDA is not able to cross the BBB. It can be unilaterally injected into the caudate-putamen (striatum) [92, 93], where it promotes prolonged and progressive retrograde degeneration of the nigrostriatal neurons, or into the substantia nigra (SN) or median forebrain bundle (MFB), which is more invasive and can promote faster neurodegeneration [94]. The dorsomedial region of the striatum is innervated by neurons originating from the SNpc, ventral tegmental area (VTA), frontal cortical area, and limbic system. 6-OHDA lesions to the dorsomedial striatum have a general effect on locomotion, and amphetamine and apomorphine induce rotational behaviour in unilaterally lesioned rats, whereas lesions to the ventrolateral striatum have marked effects on movement onset, sensorimotor orientation, and fine motor behaviour, all of which are typical signs of PD [95]. It is to be noted that 6-OHDA solutions must be prepared immediately prior to surgery since it is light sensitive and can oxidize after dilution. The vehicle used to dissolve 6-OHDA is sterile saline (0.9%) containing ascorbic acid (0.01-0.3%). Ascorbic acid is needed to stabilize 6-OHDA, as it prevents oxidation of 6-OHDA to an inactive form. In addition, a noradrenergic reuptake inhibitor, like desipramine, is often systemically injected in order to protect against damage to the noradrenergic system in the brain. However, PD is now recognized as a multisystem disorder and degeneration of the noradrenergic system in PD patients occurs and is associated with PD symptom progression, so some of the most recent studies have opted against the protection of noradrenergic terminals [22, 23, 96, 97].

A neuroimaging study of the 6-OHDA rat model confirmed that degeneration of dopaminergic neurons, evaluated by 4-[123I]iodophenyltropane (β-CIT) SPECT, is accompanied by acute microglial activation in the SN, suggesting that degeneration of nerve terminals is not a trigger for microglial activation, whereas degenerating cell bodies strongly trigger inflammatory cell infiltration [98]. A main advantage of the 6-OHDA model is that a single administration is sufficient to induce motor symptoms, like bradykinesia, which can be reversed by levodopa, and dysfunction in cognition, enteric nervous system and day/night activity have been reported [99-101]. In addition, long-term treatment with levodopa also promotes involuntary movements similar to levodopa-induced dyskinesia, a main side effect of levodopa therapy in PD patients, giving researchers the opportunity to investigate this debilitating condition. A major disadvantage is the invasive surgery which promotes a neuroinflammatory response independently of 6-OHDA effects. Some researchers use the contralateral side of the 6-OHDA model as a ‘control’, however due to possible compensatory responses observed in this model and intra-hemispheric connections complicating the matter, this may not be ideal, and an injection of saline in additional animals may provide a more appropriate control [102]. Furthermore, the model is limited by a lack of α-syn pathology, which is a hallmark of PD. Tables **2** and **3** describe the PET studies and imaging biomarkers used in the 6-OHDA model to study disease mechanisms and the effects of therapeutic interventions in the last 5 years.

#### 4.1.2 1-Methyl-4-phenyl-1,2,3,6-tetrahydropyridine (MP-TP)

After i.v. systemic administration, MPTP crosses the BBB and is converted to MPP^+^ by MAOB in astrocytes, which has a high affinity for DAT. Similar to 6-OHDA, MPP^+^ promotes oxidative stress *via* mitochondrial complex I inhibition [103]. Despite several descriptions in the literature using different administration pathways, the most common and reproducible way remains i.v. systemic administration. MPTP is mainly used in non-human primates and in mice as rats do not metabolise MPTP to MPP^+^.

MPTP affects other brain areas as well as the nigrostriatal system, including the locus coeruleus, which is also subject to neurodegeneration in PD. A disadvantage is that in order to consolidate the model, it requires several injections, which is more stressful for the animal [104]. If sedatives or mild anaesthesia are used for the repeated injections, these may confound the interpretation of the data. Furthermore, MPTP can induce parkinsonism in humans, so there is a risk of contamination during the administration [105]. Also, systemic administration of MPTP can only be used for studies that focus on bilateral neurodegeneration [106]. In most PD patients, disease onset is clinically asymmetric, so the unilateral 6-OHDA model can better represent the human condition, especially in the early stage of the disease [107]. The non-human primate MPTP model can spontaneously recover from motor symptoms without intervention, so PET can be used to study brain plasticity and compensatory mechanisms involving the dopaminergic and serotoninergic systems between baseline, early symptomatic, full symptomatic and recovered conditions. [^18^F]FDOPA and [^11^C]raclopride have been used to evaluate aromatic amino acid decarboxylase (AAAD) and D2 availability changes, while [^11^C]DASB and [^18^F]MPPF have been used to examine serotonin transporter and 5HT_1A_ function during recovery. Striatal DA D2 receptors are upregulated after MPTP but normalise during recovery. Reduced striatal AAAD activity parallels severity of motor symptoms. [^11^C]DASB binding at baseline rises with motor score in the parkinsonian condition as a compensatory mechanism [108]. Despite some spontaneous recovery, the non-human primate model has been used to assess the effects of experimental therapies, including electroconvulsive therapy and retinal human epithelial cell injections [109, 110]. The use of the MPTP neurotoxin in mice, particularly in transgenic and genetic models, allows the study of the interaction between gene deficits and neurotoxins helping to determine which genes may increase vulnerability or resistance to MPTP, for example, Fas-deficient lymphoproliferative mice have increased vulnerability [111-115]. Tables **4** and **5** describe the PET studies and imaging biomarkers used in the MPTP model to study disease mechanisms and effects of therapeutic interventions in the last 5 years.

#### 4.1.3 Rotenone

Rotenone is a pesticide used as an insecticide in vegetable gardens and is a mitochondrial complex I inhibitor that can cross the BBB and cause oxidative stress, particularly in DA neurons [158, 159]. The damage to mitochondrial complex I promoted by rotenone has been used to develop PET probes ([^18^F]BCPP-EF) as markers of mitochondrial M1 function in neurodegenerative diseases [160]. Due to its non-specific action, the rotenone model has a general system and organ effect, which leads to high mortality and requires careful titration. Similar to MPTP, it is a model used for bilateral dopaminergic cell loss studies, and it has been shown to induce α-syn fibril formation [158]. In the last 5 years, only two PET studies have used rotenone as a PD model. The studies focused on developing an adenosine A2A receptor (A2AR) tracer, with antagonists of A2AR, to evaluate the A2AR upregulation observed in the striatum of PD, which appears to be related to dyskinesia. The authors imaged rotenone-treated mice with [^18^F]FESCH, and no significant difference in the striatal A2AR density between rotenone-treated mice and controls was detectable by PET imaging or immunofluorescence staining. These results indicate that the rotenone model does not reflect the upregulation of striatal A2AR in PD [161]. On the other hand, [^18^F]FLUDA looks to be a more promising tracer than [^18^F]FESCH for evaluating A2AR availability in neurodegenerative diseases [162].

#### 4.1.4 Lipopolysaccharide

LPS PD model is based on the LPS endotoxin released by the outer membrane of Gram-negative bacteria. LPS can be injected into brain areas or intraperitoneally. It is a powerful trigger of inflammatory processes and peripheral administration of LPS in mice induces astrocyte and microglia activation, as well as cyclooxygenase-2 (COX-2), inducible nitric oxide synthase (iNOS) and pro-inflammatory cytokine expression in the brain. This inflammatory response leads to a progressive dopaminergic neurodegeneration model [163]. As a disadvantage, this general inflammation is non-specific and can affect other brain areas [163]. Furthermore, there is a chance of also causing endotoxic shock, which increases the risk of death [164]. Considering the well-known characteristic of neuroinflammatory activation, this model is widely used to develop and evaluate markers of TSPO expression, as well as other neuroinflammatory markers, such as CSF1R [165]. 2 days after 6-OHDA striatal injection, LPS has been injected i.p to induce inflammation. [^18^F]FEPPA TSPO imaging was performed before and 4 hours after LPS administration and revealed that the expression of inflammatory cytokines and the TSPO PET signal increased in parallel [119]. Uptake of three TSPO tracers, [^18^F]GE-180, [^18^F]DPA-714, and [^11^C]PK11195, was compared 3 days after LPS injection into the striatum. The second-generation TSPO-PET tracer [^18^F]GE-180 detected neuroinflammation that was not observed with either [^18^F]DPA-714 or [^11^C]PK11195 [166]. In more recent studies, a CSF1R PET ligand has been assessed using the LPS model [165].

The intrastriatal LPS model has also been used to evaluate cannabinoid receptor type 2 (CB2R) expression, a promising target for the diagnosis and therapy of central nervous system (CNS) inflammatory responses. PET and *in vitro* autoradiography with [^11^C]A-836339 were performed but showed a lack of specific uptake suggesting this radiotracer as not suitable for imaging CB2 receptors expressed under neuroinflammatory conditions [167]. The P2X7 receptor, an adenosine triphosphate (ATP)-gated purinoreceptor, has also emerged as a key player in neuroinflammatory processes, being expressed by M1-activated microglia and suggesting a role in microglial activation. The LPS model has been used to validate [^18^F]JNJ-64413739 as a potential tracer for P2X7 [168].

Rats that had systemic administration of LPS followed by microinjection of sodium nitroprusside (SNP) to induce reactive oxygen species (ROS) were imaged with [^11^C]hydromethidine ([^11^C]HM), a potential PET marker of ROS in the brain. The tracer showed good brain penetration and increased retention of radioactivity in animal models of oxidative stress [169].

Sphingosine-1-phosphate (S1P) is a potent bioactive lipid mediator that acts as a natural ligand upon binding to five different receptors located in astrocytes, oligodendrocytes, microglial and neuronal cells. Recently, global activation of these receptors by FTY720 (fingolimod) and the selective agonist SEW2871 have been suggested to provide neuroprotection in an animal model of PD [170]. The LPS-induced murine neuroinflammation model was used to evaluate [^18^F]12b toward S1PR1 by *in vitro* autoradiography [171].

The LPS model has been used to determine whether antipsychotic medication affects microglia *in vivo*. TSPO expression was evaluated by autoradiography with [^3^H]PBR28 after haloperidol therapy (0.05 mg and 2.5 mg slow-release pellets over 2 weeks). The authors reported that haloperidol at either dose did not alter microglial measures and TSPO expression [172]. Another study focused on the P2X7 receptors as a target for PD therapy and evaluated the effects of the P2X7 antagonist, JNJ-55308942. Two days after a single systemic LPS injection (0.8mg/kg, i.p.), the authors assessed *ex-vivo* brain occupancy at 2h post JNJ-55308942 (30 mg/kg, oral) with [^3^H]JNJ-54232334, and reported attenuated LPS-induced microglial activation [173].

### 4.2 α-synuclein Models

α-syn is an important protein for synaptic vesicle recycling, and its abnormal aggregation may be responsible for PD development. α-syn models are based on targeting the SN or striatum of rats, mice, or non-human primates, and promoting overexpression and aggregation of α-syn to form Lewy Body and Lewy neurite inclusions. The levels of α-syn expression and spreading determine the disease severity [174] and elicit both reliable motor impairment [175] and non-motor symptoms [176]. The propensity of α-syn strains to aggregate is based on a number of factors, such as post-translational modifications, gene duplication and triplication-driven overexpression, single point mutations and environmental changes [177]. The popular toxin-induced model, 6-OHDA, would not be a model of choice to represent idiopathic PD due to the lack of clear evidence of α-syn accumulation. There are several ways to promote α-syn aggregation: 1) genetic modification, 2) proteasome and lysosome inhibition, 3) AAV, and 4) α-syn preformed fibrils.

#### 4.2.1 Genetic Models

During the last 25 years, there have been several important discoveries of genetic risk factors for PD. First-degree family members of affected patients were reported to have a 2- to 3-fold increased risk of developing the disease compared to subjects in the general population or controls. Monogenetic mutations in 20 genes causing PD have now been identified [178] and, despite those mutations being rare, a genetic cause has been implicated in as many as 5-10% of the Caucasian PD population [179]. This rises to 40% in Ashkenazi Jews, who can carry both LRRK2 and GBA mutations [180, 181], and North African Arabs, 30% of who carry the LRRK2 G2019S mutation [182]. GBA mutation frequency in the European non-Ashkenazi Jewish population is 2.9-12%, whereas in the European Ashkenazi Jewish population, it is 10-31% [7].

Genome-wide association studies (GWAS) have suggested an increased risk of developing PD in persons with a range of susceptibility genes or a family history of PD or tremor. Six genes have been proposed to mediate autosomal dominant forms of PD: SNCA, LRRK2, VPS35, EIF4G1, DNAJC13, and CHCHD2 [176]. SNCA, which encodes the protein α-syn, was the first gene to be associated with autosomal dominant parkinsonism, but polymorphisms also increase the risk of sporadic disease. Mutations in LRRK2 are the most common mutation in dominant familial PD, and Pten-induced kinase 1 (*PINK1*) and *PRKN* are associated with recessive PD and are associated with mitochondria dysfunction and failure of ubiquitin ligation. The highest genetic risk factor for developing sporadic PD is mutations in GBA, which encodes lysosomal enzyme GCase, suggesting that the use of animal models expressing GBA mutations might be most relevant to study mechanisms of idiopathic PD and for trialling novel therapeutic strategies [7]. A knowledge of susceptibility genes for PD has led to the development of genetically induced PD animal models. Genes involved with complex I of electron transport in mitochondria, oxidative stress, and protein dysfunction are the targets of these models. Some models fail to induce DA neuron loss, the major hallmark of PD, despite the models being able to induce α-syn aggregation and produce Lewy body-like inclusions. Those genetic models may be a good option for novel early neuroprotective trials, before the loss of neurons.

The GBA gene mutation is also the causative gene of autosomal recessive Gaucher disease, a disease that carries a high risk of developing PD, and mouse models of Gaucher disease are being used to study PD. However, 300 potentially pathogenic mutations in the GBA gene have now been uncovered, and their link to sporadic PD is unclear [7]. Mutations in the *GBA* gene may lead to loss of GCase activity and lysosomal dysfunction, which may impair α-syn metabolism. An inverse correlation has been observed between GCase activity and α-syn accumulation in *GBA*-PD and sporadic PD brains. Certain mutations cause GCase to be misfolded and retained in the endoplasmic reticulum, which may contribute to neurodegeneration due to the activation of stress responses, including the unfolded protein response. In addition, GCase deficiency has also been associated with mitochondrial dysfunction and neuroinflammation, mechanisms involved in PD pathogenesis [183, 184]. GBA1 D409V knock-in mouse astrocytes showed clear impairment in lysosomal morphology and function, which was normalized by inhibition of LRRK2 kinase activity. This correlation indicates intracellular crosstalk between GCase and LRRK2 activities in astrocytes [185]. However, wild-type GCase overexpression in mice revealed lower lipid-rich aggregates accumulation and amelioration of PD-like phenotypes [186]. To test the hypothesis that the gene mutations can be related to α-syn spread, α-syn preformed fibrils were injected into the olfactory bulb of mice carrying GBA D409V+/- and ATP13a2; these mutations did not exacerbate behavioral impairments or histopathology (α-syn, LAMP2, and Iba1) when compared to their wild-type littermates [187], reinforcing the unclear role that GBA has in PD development.

CRISPR/Cas9-targeted large animal (pigs and monkeys) specific gene-editing has uncovered important pathological events that resemble neurodegeneration in PD brains [188] that could not be produced in small animal models [189]. *PINK1* and *DJ-1* are the most targeted genes with CRISPR/ Cas9 editing and have led to classic PD syndrome and severe nigral dopaminergic neuron loss in monkeys but not in pigs [188]. The PARK1 model is based on encoding A30P, A53T, and E46K α-syn substitutions. The data regarding dopaminergic neuron loss is controversial [190]. There are descriptions of decreased DA levels and TH, with and without dopaminergic neuronal loss. Previous data revealed controversial data for LRRK2 models. Overexpression of LRRK2 promotes mild or no disruption of nigrostriatal DA neurons. There is an age-dependence between progressive motor deficit and mild reduced striatal DA release. KO LRRK2 mice revealed no neurodegeneration but showed changes in neuronal morphology and a-syn aggregation. Rats overexpressing human LRRK2 p.G2019S performed significantly worse on the rotarod than their non-transgenic littermates at 6 months of age but performed normally on other motor tests. PET imaging using [^11^C]MP (methylphenidate) as a marker of DA transporters, [^11^C]DTBZ (dihydrotetrabenazine), [^18^F]FDOPA and [^11^C]raclopride performed at 12 months did not recapitulate prior studies in human LRRK2 mutation carriers, suggesting that LRRK2 p.G2019S rats do not develop overt neurodegeneration and only develop behavioural abnormalities [191]. On the other hand, longitudinal [^11^C]PBR28 PET imaging revealed that a single LPS treatment in LRRK2 p.G2019S caused inflammation in the brain over 10 months, while in the non-transgenic model, the increase was not significant. No dopaminergic degeneration was observed. Translationally, this implies that repeated exposure to inflammatory triggers may be needed for LRRK2 mutation carriers to develop active PD [192].

*In vitro* and *in vivo* models have been developed that support the role of PINK1 in synaptic transmission, particularly affecting dopaminergic neurons. It is of paramount importance to further define the role of PINK1 in mitophagy and mitochondrial homeostasis in PD pathogenesis in order to delineate novel therapeutic targets. KO PINK1 mice revealed age-dependent DA depletion and impaired motor activity without neuron loss. There was no Lewy body formation and no neurodegeneration. On the other hand, overexpression of α-syn in these animals caused neurodegeneration. Compound heterozygous or homozygous parkin gene mutations are associated with early PD and cause dysfunction of the ubiquitin-proteasome system (UPS). They also lead to impaired mitophagy, accumulation of protein, and mitochondria dysfunction. Parkin KO animals revealed DA decrease, and again no neuronal loss was observed. Protein deglycase (DJ-1 gene - PARK7) KO mice revealed DA decrease, locomotor impairment without SNpc but with VTA neuron loss. Motor behavioural deficits and progressive bilateral degeneration were reported, but no Lewy body formation was described. In mice, DJ-1 mutations could be a model for prodromal PD studies [69]. DA receptor expression and DA levels in parkin-deficient mice were evaluated by *ex vivo* autoradiography, using [^11^C]DTBZ, [^11^C]SCH23390, [^11^C]raclopride, L-[β-^11^C]DOPA and [^11^C]β-CFT. Parkin deficiency was associated with considerable upregulation of DA (D1 and D2) receptor binding *in vivo* in the striatum and increased DA levels in the midbrain. A clear decrease in endogenous DA release after methamphetamine challenge was also reported [193].

The transgenic MitoPark mouse PD model combined with longitudinal [^18^F]-FE-PE2 PET (at weeks 6, 10, and 20) was used to investigate the long-term effects of voluntary exercise on motor behaviour and the DA system. The study revealed a transient but significant increase in striatal DAT binding at 10 weeks in exercised mice, which was no longer apparent at 20 weeks, probably due to the severity of the phenotype of this model [115]. Cell-based drug delivery therapy was evaluated in transgenic Parkin Q311(X)A mice, where GDNF-transfected macrophages were administered through intravenous, intraperitoneal and intrathecal routes. GDNF-transfected macrophages administered through the intrathecal route provided significant increases of GDNF levels in different brain sub-regions, including midbrain, cerebellum, frontal cortex, and pons. The biodistribution was evaluated by 64Cu-labeled macrophage PET, and revealed a preferential transport and accumulation of transfected macrophages in the inflamed brain in PD animals [194].

#### 4.2.2 Proteasome Inhibition

Years after the discovery of α-syn as a core component of Lewy bodies, studies of protein clearance increased, including investigations of the deficiencies in proteasome activity, mitophagy, ubiquitination and autophagy. Specifically, the UPS received attention as it is the proteolytic route for small, misfolded, damaged and short-lived proteins like misfolded monomeric α-syn, while the autophagy-lysosome pathway (ALP) was studied for its potential role in the degradation of large and long-lived cellular components, including protein aggregates and organelles [195, 196]. An α-syn transgenic mouse model was used to demonstrate that the UPS is the main degradation pathway for α-syn. An increased load of aggregated α-syn led to the recruitment of the ALP [197]. Interest in the UPS was kindled by discoveries of UPS mutations in cases of familial PD, including *parkin* and *UCHL1* [198-200], detection of UPS components in Lewy bodies [201], and decreased proteasomal activity and expression in SN of PD patients [202]. The classic models, like 6-OHDA and MPTP, did not demonstrate significant effects on protein aggregation and the UPS, so newer models were needed to study this aspect of PD. Lactacystin is a proteasome inhibitor that is naturally synthesized by bacteria [203]. By inhibiting the proteasome, the degradation of several proteins is blocked, which has been shown to seed the formation of cytoplasmic aggregates containing α-syn and ubiquitin [204]. In early studies, intranigral and striatal injections of lactacystin were shown to induce motor deficits, dose-dependent nigral degeneration and α-syn inclusion bodies in rats [202, 205, 206]. A study reported decreased ipsilateral striatal [^18^F]DTBZ PET binding to the vesicular monoamine transporter 2 (VMAT2) two and three weeks after lactacystin injection to the MFB and confirmed the loss with immunohistochemistry of TH and VMAT2 [205]. Similar findings were reported in a study [207], including decreased ipsilateral [^11^C]DTBZ PET binding and TH immunoreactivity in the SN.

The motor dysfunction induced by lactacystin can be reversed with apomorphine and levodopa [202, 208], and the model replicates several features of human PD, including reduced proteasomal enzyme activity, α-syn aggregates, dopaminergic degeneration and neuroinflammation [209]. As aging is a predisposing factor for PD, the use of adult mice also showed increased sensitivity to nigral lactacystin injection compared to young mice, including motor impairment, nigral α-syn accumulation and DA cell loss; striatal DA decreased fiber loss and widespread inflammation [210]. The UPS inhibition model has only once been trialled in non-human primates using a systemic protocol proposed for rodents using the proteasome inhibitor (PSI) [211]. However, systemic PSI administration to cynomolgus monkeys failed [212], similarly to the later failed attempts of a rodent systemic PSI model [213] presumably owing to route-dependent bioavailability [214]. Further issues with the use of the PSI model were associated with the use of ethanol as a vehicle, which itself caused DA neuron degeneration [215].

More recent studies involving a large animal, the Göttingen minipig, have been conducted using acute MFB injections [53] and chronic intracerebroventricular (ICV) injections of lactacystin [54], and both studies have found motor impairment and decreased [^11^C]DTBZ PET binding in the ipsilateral striatum using PET. The chronic study followed the minipigs for 6 months and observed a reversal in striatal DBTZ binding, indicating compensatory changes to the repeated low doses of lactacystin. This chronic ICV model also led to neuroinflammation and impacted serotonin and noradrenaline neurotransmission so it may be a model for non-dopaminergic aspects of PD [54].

The limitations of the proteasome inhibitor models include the failure of the systemic models and a general cell toxicity, since it is not specific for dopaminergic cells. The translatability to human PD has also been questioned since the decreased activity of proteasomes could be a consequence rather than a cause of the neurodegeneration and α-syn accumulation. A review that highlighted the dysfunction of cellular proteostasis in PD concluded that there is no evidence of defective proteasome activity in unaffected brain regions, and in some areas, increased activity is reported. So, these studies suggest that reduced proteasome activity is specific for certain brain regions, like SN. Consistent with these findings, *in vivo* studies showed reduced proteasome activity in SN after rotenone [158] and continuous MPTP administration [216]. The UPS impairment caused by MPTP was alleviated in mice lacking α-syn suggesting that it increases the detrimental effects of MPTP on the UPS [217]. These models open the door for interesting studies combining proteasome inhibition with overexpression of α-syn [218] or LPS-induced neuroinflammation [219].

#### 4.2.3 Recombinant Adeno-Associated Viral Vectors

Recombinant adeno-associated viral vectors (rAAV) can be used as a vehicle to deliver a specific gene, such as gene therapy or a gene associated with a disease. When delivered to the host tissue, the viral capsid proteins will mediate delivery into the nucleus, and the single-stranded DNA from the rAAV can be converted into double-stranded DNA by the host DNA polymerase before it is transcribed into mRNA [220]. Most often, it is not integrated into the host DNA and therefore does not cause gene defects by random/off-target insertion. rAA*Vs* can transduce both dividing and non-dividing cells, in contrast to lentivirus, which can only transduce non-dividing cells [221]. The rAAV particles are smaller than lentivirus and can, after stereotaxic delivery, spread to a larger area [222] compared to lentivirus that will only spread a couple of millimetres [223]. The small size also allows for a higher virus concentration/titer in the injected volume. rAA*Vs* can be used to overexpress wild-type or mutated (*e.g*., A53T or A30P) α-syn.

Local injections in SN of rAAV can infect nigral dopaminergic neurons [224] with a ~90% transduction of TH-positive cells in rats and human primates [225]. Nigral injection of wild-type or A53T human α-syn rAA*Vs* into rats causes PD pathology in the form of progressive development of inclusions and swollen dystrophic neurites of dopaminergic cells, 30-80% nigral dopaminergic cell loss, 40-50% reduction of DA and TH in the striatum, and decreased motor function [225]. Injection of rAAV human wild-type α-syn into the SN of rats showed profound motor impairments and progressive loss of dopaminergic nigral neurons, when including an enhancer and using a different promotor [226]. rAAV human wild-type and A53T injections into the SN of mice lead to motor impairments and dose-dependent neurodegeneration in the nigra (up to 82%) correlating with a reduced striatal TH immunoreactivity [227].

Other studies have also found motor impairments after rAAV human wild-type α-syn injections to the SN of rats along with loss of striatal terminals but not nigral cell loss, in opposition to earlier studies. This was accompanied by aggregations of phosphorylated α-syn in terminals and *in vivo* ipsilateral decreased binding of [^11^C]DTBZ to VMAT2 in dopaminergic terminals [228]. This study suggested that α-syn pathology can start in the axons and dendrites and later spread to the soma. Further investigations in this model showed increased immune activation using [^3^H]PK11195, and reduced DA D2/3 expression with [^3^H]raclopride [229].

In non-human primates (marmosets), rAAV wild-type or A53T human α-syn injections into the SN revealed that α-syn was distributed by anterograde transport from the soma to axonal and dendritic projections. Swollen dystrophic neurites and α-syn positive cytoplasmic inclusions were observed along with motor impairments and a 30-60% loss of TH positive neurons and innervation to caudate putamen [224].

Different AAV capsid serotypes have different transduction volumes and the neuron affinity also depends on the promoter, coat binding proteins and neuron cell surface sugars and receptors [230, 231]. The rAAV 2/5 vector carrying the human α-syn A53T gene has shown transduction of SN dopaminergic neurons in non-human primate, minipig and rodent though variable onset and peak of transgene expression, and different levels of motor impairments were observed [224, 232-234]. Interestingly, transduction of glial cells and GABAergic medium spiny neurons were also found in the marmosets [232] while a minipig study showed only medium spiny neuron and glial transduction of the AAV2/5 after injection to the SN [52]. Careful considerations should, therefore, be made when selecting AAV expression cassette and serotype as outcomes can vary between species, animal age, injection site and neuron subtype of interest. Decreased [^11^C]raclopride was observed in KO α-syn mice compared to wild-type mice and an accelerated murine model of synucleinopathy (M83), suggesting a D_2_ receptor expression modulation associated with α-syn [235]. Crabbe *et al.* also observed a higher [^18^F]MPPF uptake in M83 *vs*. WT and KO mice, which could indicate overexpression of 5-HT_1A_ receptors due to α-syn aggregation. In an α-syn rat model, *in vivo* [^18^F]FDG imaging showed increased glucose metabolism in the SN and corticostriatal regions, which was also correlated with motor impairment and TSPO neuroinflammation in the SN by *in vitro* [^18^F]DPA-714 autoradiography [236]. The advantages of viral vector-mediated models are that they target the nigro-striatal system and there is the possibility to induce the pathology during adulthood and to adjust the dose/expression levels of α-syn [237]. Since this model promotes α-syn pathology, it can aid in the development of treatments targeting α-syn toxicity. However, for gene therapy, the viral vector-based models can cause an unfavourable interaction in subsequent viral transductions altering transfection and the reliability of experimental results [238]. In a rat model of PD induced by unilateral injection of AAV serotype 9 (AAV9) expressing A53T α-syn (AAV9-A53T-α-syn), the effects of 8 weeks of treatment with Fasudil (5 mg/kg/day), a Rho-associated protein kinase 2 inhibitor, were evaluated with the VMAT2 tracer, [^18^F]DTBZ. Fasudil significantly enhanced the radioligand uptake in the injected striatum of the rat model. In addition, there was an improvement of motor deficits, evaluated by cylinder and rotarod tests, and Fasudil could promote the autophagic clearance of α-syn by Becline 1 and Akt/mTOR pathways [239].

#### 4.2.4 Preformed Fibrils

More recently, PFF models have been developed from monomeric recombinant α-syn proteins. PFFs are injected into the cerebral dopaminergic system, where they can seed/trigger endogenous α-syn to misfold and form inclusions similar to Lewy bodies. This was inspired by PD patients receiving dopaminergic grafts, where Lewy body-like inclusions were found in the grafts years after transplantation, showing that α-syn pathology can spread from host to graft [212, 240]. This led to the theory that α-syn can be released from one cell and taken up by another in a prion-like manner, seeding the misfolding of endogenous α-syn and causing aggregation in the new cell [241]. PFFs or brain lysate/homogenate from PD patients are injected into animals to study the seeding, spread, aggregation, phosphorylation and ubiquitination of the fibrils. Cerebral injections of brain homogenates with pathological A53T α-syn or synthetic α-syn into mice have led to the development of Lewy body-like inclusions, the spread of pathological α-syn to other brain areas, dopaminergic nigral neurodegeneration, and accelerated death [242, 243]. Similar results have been found in rats with the spreading of pathological α-syn (pSer129) to cortical areas, the olfactory nucleus, amygdala, thalamus, striatum and SN along with neurodegeneration of dopaminergic cells in the SN [242, 244, 245]. PFF inoculation can start an immune response as MHCII-positive ramified cells are found in the brain [246, 247]. In non-human primates, striatal or nigral injections of pathological α-syn from Lewy body brain extracts resulted in a loss of dopaminergic cells in the striatum and SNpc [248], and striatal injection of synthetic α-syn caused the spreading of pathological α-syn to other brain areas and Lewy body-like inclusions in the SNpc [249]. These findings show that pathological α-syn can propagate from the striatum to the SN. In contrast to a general expression, using transgenic models, or a local expression, using viral vectors, α-syn PFFs can seed to interconnected brain areas [250].

For the extensive testing of an α-syn PET ligand, a progressive model of α-syn accumulation would be the ultimate choice. One such model, the preformed α-syn fibril rodent model of PD, has shown a 3-fold increase of phosphorylated α-syn in motor cortex 6-22 weeks post-PFF injection and spreading of α-syn aggregates [247], and would be appropriate for the evaluation of chronic changes and correlation with symptom severity. An alternative approach for the initial screening of lead compounds would be the acute local injection of fibrils into the cortex rather than the nigrostriatal system, an approach currently being trialed in Danish farm pigs [251]. This rapid approach would allow a fast screen of potential ligands, but has the disadvantage of the ligands not being trialled in a classical PD-associated brain regions or in a relevant progressive model. A potential radioligand for α-syn, [^11^C]MODAG-001, showed an excellent ability to penetrate the mouse brain. Metabolic degradation was present, but the stability of the parent compound improved after selective deuteration of the precursor. (d_3_)-[^11^C]MODAG-001 binding was confirmed in fibril-inoculated rat striata using *in vivo* PET imaging. [^11^C]MODAG-001 seems to be a promising tracer for striatal α-syn-inoculated rats, which revealed a very high affinity towards pure α-syn fibrils and only a moderate affinity to hTau46 fibrils. However, this tracer also binds to β-amyloid fibrils [83].

## 5 BRAIN-FIRST OR BODY-FIRST HYPOTHESIS: ANIMAL MODELS

Animal models have been extremely useful for studying hypotheses about α-syn initiation and propagation. Specifically, the body-first models could be important models to study when investigating gut symptoms, pathology and alterations to the gut microbiota, and furthermore, they can be useful as prodromal models of PD for testing early disease-modifying therapies and investigating biomarkers for PD. A detailed review on the brain- and body-first animal models has recently been published [252].

It still remains an unanswered question of where and how PD is initiated [253]. Braak *et al.* proposed a gut-first hypothesis where α-syn pathology may be initiated in the enteric nervous system from where it spreads through parasympathetic and sympathetic connections to the dorsal motor nucleus of the vagus nerve (DMV). This hypothesis was based on brain pathological staging of sporadic PD brains, where initial pathology was observed in the DMV and anterior olfactory nucleus [174, 253]. Several studies have now shown that α-syn can spread transcellularly between the periphery and CNS and between brain regions. The peripheral-onset hypothesis proposes that aggregated α-syn transmission starts in the enteric nervous system and propagates *via* cell-to-cell transmission through sympathetic and parasympathetic nerves to the DMV and intermediolateral nucleus into the CNS [254]. Though it is still debated [255], this theory is supported by animal studies showing that intragastric administration of rotenone can induce α-syn accumulation and spread to the CNS through autonomic nerves [256]. Furthermore, propagation of pathology *via* the vagus nerve has been proposed by several studies [257-259], but the hypothesis still does not explain the heterogeneous phenotype of PD, and not all patients follow the staging scheme proposed by Braak with a caudo-rostral progression [260].

In the last years, a new hypothesis has been proposed, called the α-syn origin and connectome (SOC) model, that uses both the initial anatomical location of α-syn inclusion and the connections through which α-syn propagates to explain the disease heterogeneity [11]. The theory proposes two PD subtypes and is supported by multimodal imaging data [261]. In brain-first subtypes, the initial α-syn pathology arises unilaterally in the CNS, often in the amygdala or olfactory bulb, from where it spreads primarily through ipsilateral connections to the SN and the DMV. This leads to an asymmetric spread and is, at the time of diagnosis, dominated by a low burden of α-syn, motor asymmetry, slow progression to dementia, few autonomic symptoms, and no prodromal rapid eye movement RBD. The model described above is the most commonly used for the brain-first approach. In the body-first subtypes of PD, the onset pathology arises in the peripheral nervous system, from where it propagates *via* bilateral vagal connections to the DMV, locus coeruleus and SN, and further to the whole brain. This subtype has a long prodromal phase where non-motor symptoms, like constipation, RBD and hyposmia, appear prior to more symmetric motor symptoms. Due to the larger burden of α-syn *via* bilateral transmission at the onset of motor symptoms, this model predicts a faster progression to dementia [11].

In the last decade, a lot of work has been done with animal models that in principle mimic the brain-first subtypes as there is an injection of α-syn pathology at one site in the brain, as described above in this review. Recently, this focus has shifted to also include seeding of α-syn pathology in the gut to mimic the body-first subtypes [262, 263]. Bidirectional gut-to-brain propagation of α-syn following seeding of PFFs in the duodenum has been shown in the bacterial artificial chromosome (BAC) transgenic rat model. The propagation was documented through the vagus to the DMV and through the sympathetic connectome to the celiac ganglion and intermediolateral nucleus of the spinal cord [262].

In baboons, intrastriatal or enteric injections of α-syn-containing Lewy body extracts from PD patients have shown nigrostriatal lesions and instrastriatal and enteric pathology suggesting a bidirectional route of α-syn transmission [264]. However, two years post-injection, no pathology was found in the vagal nerve or the DMV, and the authors instead propose a systemic mechanism for spread [264]. Others have found only transient pathology in the DMV at earlier time-points post-injection in both wild-type and BAC transgenic mice, though the pathology did not propagate rostrally beyond the DMV [265, 266]. Based on large population studies with long-term follow up, it has been shown that complete truncal vagotomy to treat gastric ulcers reduced the risk of PD [259, 267], and a recent animal study also showed that vagotomy protected against neuropathology [265] and behavioural deficits otherwise induced by pathological α-syn injected in the gut [263]. These findings support the transmission of pathology through the vagal nerve.

The gut-to-brain and brain-to-gut propagations of α-syn were shown to be more robust in aged *vs*. young wild-type rats, which is in line with age being the greatest risk factor for PD [32, 268]. Similarly, aged, but not young animals, showed progression of pathology to the midbrain, resulting in decreased striatal DA and motor defects following duodenal seeding of α-syn PFFs [269]. These results underscore the need to increase the number of preclinical studies on aged animals.

## 6 FINAL REMARKS

PD is a brain disorder with multiple distinct molecular, functional and structural features, and no current animal model recapitulates all aspects of this complicated disease. However, depending on the research question and the objectives of a given study, several of the available animal models presented in this review have great utility for the study of specific disease mechanisms and trialling of symptomatic and neuroprotective agents, the study of mechanisms underlying therapy-related complications, such as compulsive behaviours and dyskinesias, and the development of disease biomarkers, including novel agents for non-invasive imaging of PD patients. Indeed, there are many considerations when performing preclinical research [270]. For any study, the choice of the best animal model is a vital step, as is the selection of the optimal behavioural tests and methods for the evaluation of particular mechanisms under investigation. Based on the literature and our own experience with PD animal models ranging from mice to non-human primates and the development of novel minipig models targeting different mechanisms, we propose the following guidelines for the optimal selection of the most appropriate PD models. Fig. (**4**) presents our suggestion for which models may be most relevant for specific research questions.

We urge researchers to be cautious over the use of classical neurotoxin models, 6-OHDA and MPTP, for testing neuroprotective efficacy of novel agents. Most putative neuroprotective drugs have undergone human clinical trials after showing protective or restorative effects in 6-OHDA and MPTP-exposed animal models. However, even though these lesion models have been valuable for testing drugs in relation to symptomatic relief, to date, they have been unsuccessful in predicting the efficacy of neuroprotective therapies for human sporadic PD [271]. For example, the discovery of the most widely used treatment for PD, levodopa, was triggered by its use to reverse the intoxication associated with reserpine. In the mid-1950s, reserpine was proposed as the first selective treatment for schizophrenia. In fact, reserpine had severe side effects, including a tendency to induce parkinsonian symptoms. On this occasion, pharmacologists described a depletion of catecholaminergic neurotransmitters. Swedish pharmacologist Arvid Carlsson decided to inject the animals with levodopa because neither noradrenaline nor DA crossed the BBB, a work that won him the Nobel Prize in 2000 [272]. Although toxin models offer important insight into the pathopharmacology of PD, the limitations of their translatability to human PD must be addressed. For example, the rapid destruction of catecholaminergic neurons does not mirror the slow progression of human PD, where pathogenesis can progress over decades. Furthermore, protein aggregation/Lewy body-like pathology is inconsistent between MPTP studies and is not present in the 6-OHDA model. The presence of Lewy body-like inclusions in monkey MPTP models has been reported, especially in aged animals [86-88]. In mice, there is an upregulation of α-syn and hyperphosphorylation of tau [89] without evidence of aggregation after MPTP [90]. Although some chronic low-dose MPTP infusion studies have reported the formation of nigral Lewy-body-like inclusions, others have failed [216, 273, 274]. Taken together, we suggest that the neurotoxin models are useful for the evaluation of symptomatic and not neuroprotective efficacy of therapies. Toxin models are also useful for assessing the efficacy of therapeutics for treating motor complications associated with chronic DA replacement therapy (fluctuating motor responses and dyskinesias).

PD is no longer considered a pure dopaminergic disorder due to the debilitating non-motor symptoms associated with the disease. In recent years, the serotoninergic and noradrenergic systems have been widely investigated for their role in sleep disturbances, depression, *etc*. Animal models of PD displaying extra-dopaminergic deficits have become important. For this reason, the 6-OHDA model of PD is now often induced without the blockade of noradrenaline transporters, under the assumption that the combined DA and noradrenaline deficit would provide an improved model. There are toxins that selectively lesion the locus coeruleus and median raphe, which could be used alongside MPTP to mimic the loss of noradrenaline and serotonergic deficits present in human PD. The lactacystin-proteasome model of PD, when administered *via* the cerebral ventricles to minipigs, has been shown to affect noradrenaline and serotonin systems as well as DA. Models of this type could prove useful for the investigation of non-dopaminergic mechanisms and non-motor symptoms, such as sleep disorders and depression and their symptomatic relief.

Models involving genetic modification, in general, do not convincingly reproduce the gamut of symptoms and signs associated with sporadic PD, and thus more studies are necessary. Experts have highlighted GBA mutation as a target to understand the disease, but its role in PD development remains unclear. An inverse correlation has been observed between GCase activity and α-syn accumulation in *GBA*-PD and sporadic PD brains. The same has been observed in GCase-deficient models. Recently, midbrain-like organoids deficient in GCase and over-expressing wild-type α-syn accumulated Lewy body-like pathology, which was absent in organoids with GCase deficiency or *SNCA* triplication alone, suggesting that impaired GCase activity promotes α-syn pathology [275]. Reduced GCase activity and protein levels have been reported in the brains of sporadic PD patients at postmortem, with the greatest reduction observed in the SNpc, but it is not the only factor responsible for PD development since some Gaucher disease patients with decreased GCase activity do not develop PD [7]. So, while there are links to prove decreased GCase activity as necessary for sporadic PD development and progression, they are clearly not sufficient. More studies are necessary, and GCase remains an important target for future studies with animal models. Only a few PET studies have been reported in transgenic models of PD, which may reflect the fact that most of the transgenic models are developed in mice, and the small size of the mouse brain is difficult to image at a high enough resolution with PET to measure function of deep brain structures. The availability of genetic models in rat and larger animal models like the minipig should lead to more functional imaging studies of genetic models.

The development of an ideal tracer for monitoring the earliest stages of disease and the protective effects of therapeutic approaches avoiding invasive interventions is an active field of research. An important requirement is that the tracer uptake should not be affected by the medications under trial, must cross the BBB, and ideally should then be washed out during PET except where retained by specific binding [27]. For example, we now know that chronic exposure to levodopa or MAO-B inhibitors can decrease the availability of dopaminergic targets and can influence the PET and SPECT signals, which can confound the interpretation of functional imaging data on disease progression at follow-up or after the intervention. It has been shown with β-CIT SPECT that agonist-treated early PD cases progress at a similar rate to placebo cohorts, but levodopa artefactually depresses DAT binding. Novel radioligands are currently being developed, and here too it is important that the animal models are selected carefully. We suggest the use of a slowly progressive model of α-syn accumulation, such as the preformed fibril model in rat [247] for the trialling of α-syn novel PET/SPECT ligands. Once validated, such a marker can give the opportunity to image the spread of α-syn *in vivo* at different points in time and to test the therapeutic potential of α-syn aggregate targeting therapies. For the ongoing search of radioligand markers of neuroinflammation, one could start with the LPS model. Although not directly a model of PD, the strong inflammatory response is valuable as an initial screen. Follow-up studies in alternative models with a more modest inflammatory response, more relevant to human PD symptoms and neuropathology, such as the 6-OHDA model or the preformed fibril model, will have high value.

## CONCLUSION

Here, we have reviewed the use of animal models of PD in scientific research, along with their strengths and weaknesses, to provide the reader an overview of which animal model of PD to use and when to use it. We argue the robust combination of appropriate animal models of PD with *in vivo* biomarkers used in PET/SPECT research. If selected appropriately, the use of animal models together with PET imaging and its ability to evaluate the function of molecular targets non-invasively and repeatedly over time and correlate changes with behavioural symptoms, fluid biomarkers and other molecular biology readouts, gives the unique opportunity to follow molecular changes at baseline, in response to acute and chronic disease stages and in response to therapy in the same animals. Not only does this comply with the Reduction aspect of the 3 Rs, but it increases the statistical strength of our investigations. Animal models are also instrumental in the development of novel PET radioligands. Over the last few years, a number of new PET tracers have been tested in the PD models that are available, but a selective marker of α-syn aggregation is still an unmet need. A tracer that aids PD diagnosis and follows its progression is still the target of studies. α-syn based PD models should ideally be used for trialling putative neuroprotective or restorative agents, while toxic lesion models have their main role in testing novel symptomatic therapies.

## Figures and Tables

**Fig. (1):**
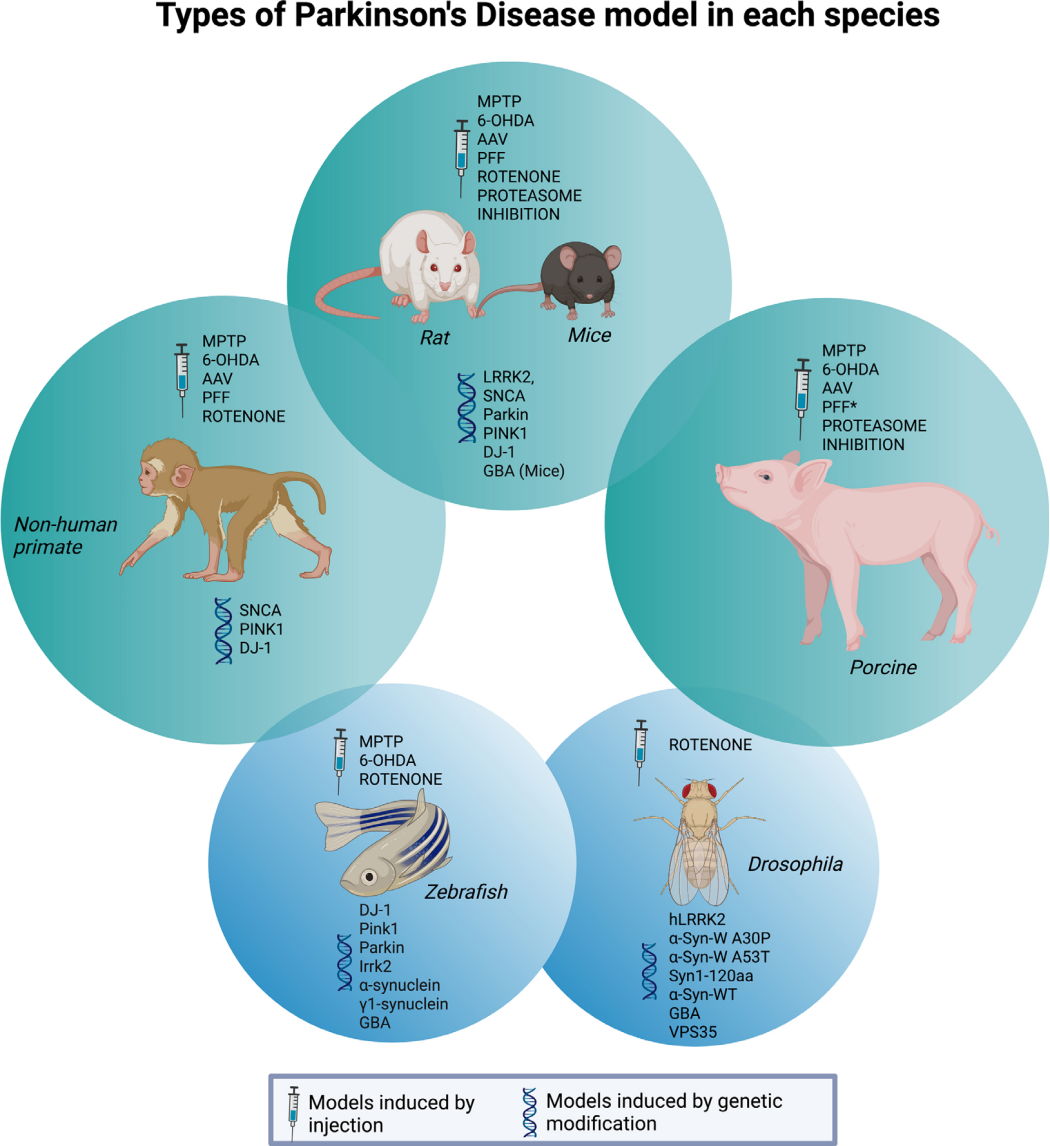
Parkinson’s disease models available in rodent, non-human primate, and pig. Zebrafish and fly models, primarily with genetic modifications, are presented as alternative models. *Preformed fibrils were used to trial potential tracers of alpha-synuclein aggregation, not to induce a Parkinson’s disease model *per se.* Created with BioRender.com.

**Fig. (2):**
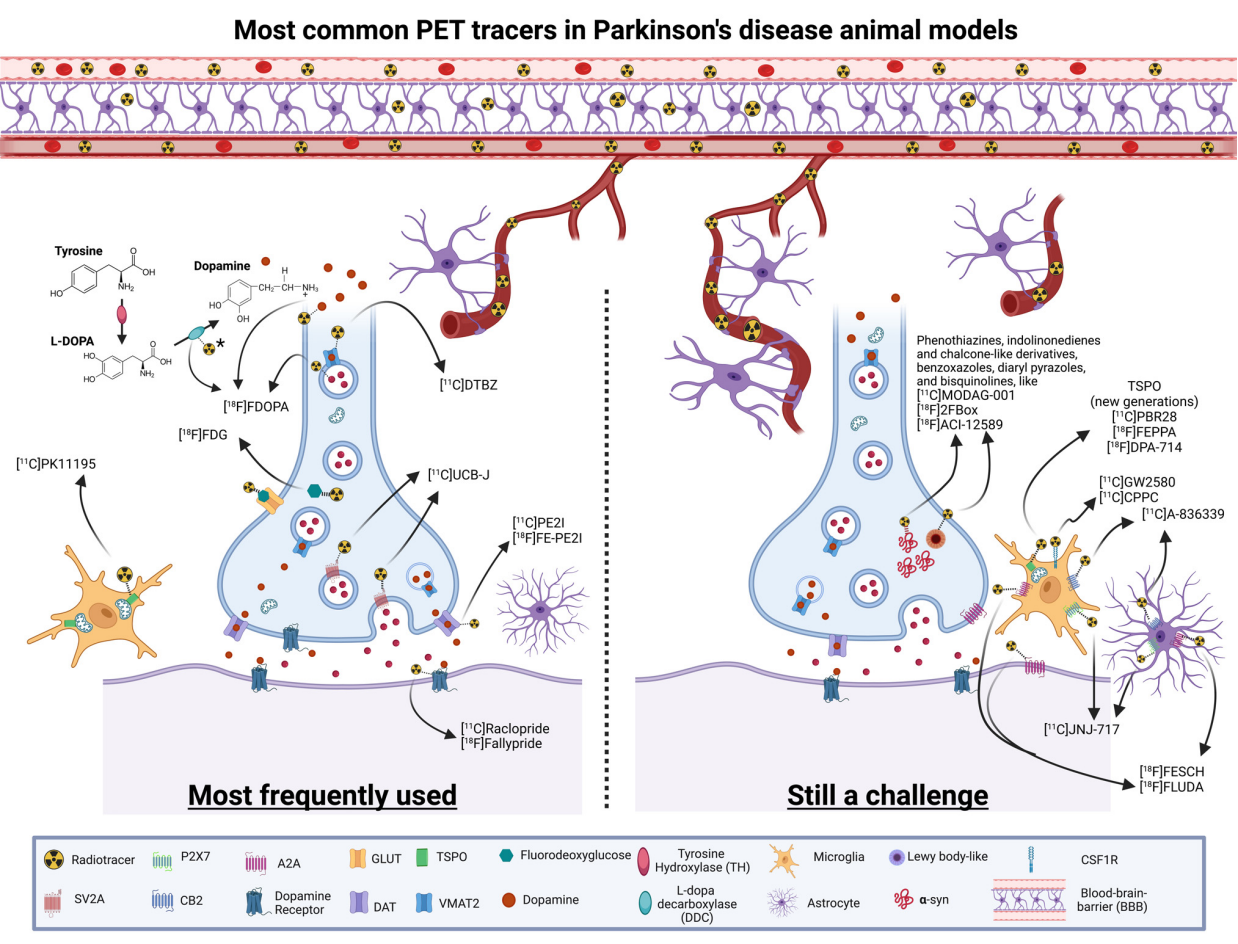
The figure illustrates the main radioligands used in preclinical Parkinson’s disease trials. It needs to be noted that an important property for successful radioligands is the ability to cross the blood-brain barrier. Some radioligands for the same target are still under development (new generations) since the data was controversial or did not show a good signal-to-noise ratio, as happened with TSPO and α-synuclein tracers. Created with BioRender.com.

**Fig. (3):**
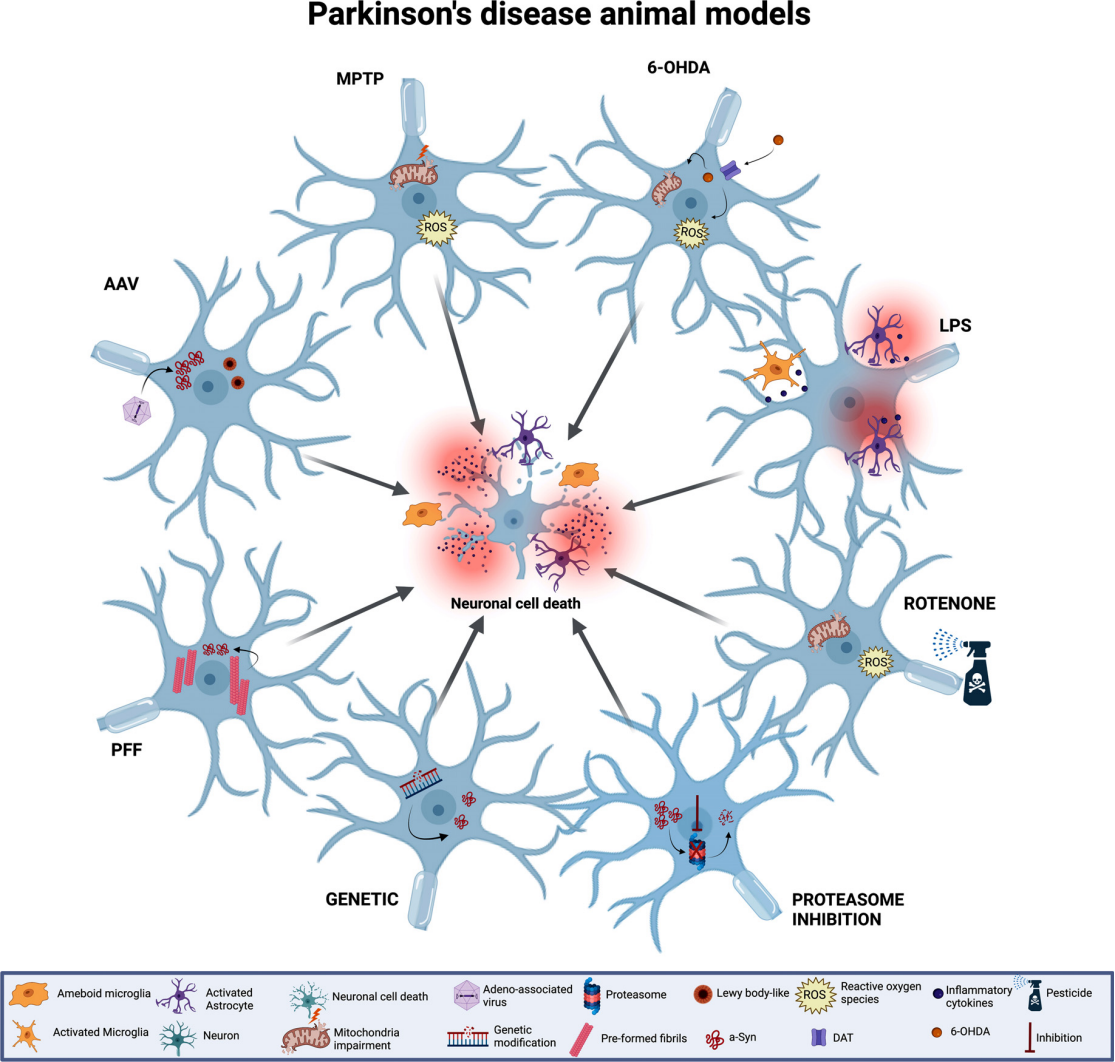
Primary pathophysiological mechanisms of the most common animal models of Parkinson’s disease. Created with Bio-Render.com.

**Fig. (4):**
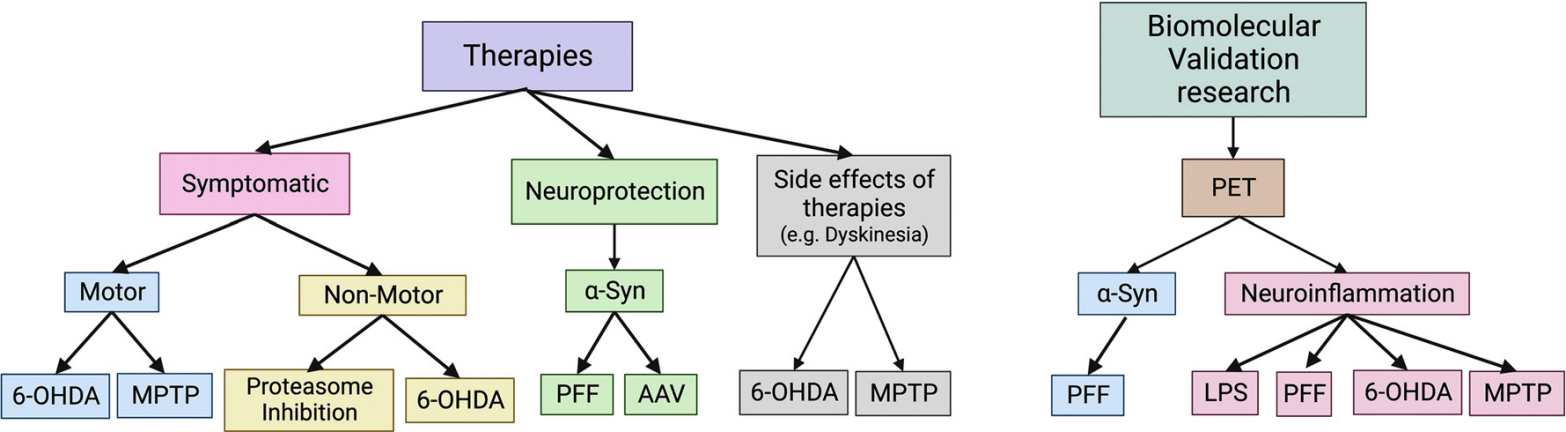
Flow chart illustrating, in short, which Parkinson’s disease animal model can be most suitable for each main research question. Created with Bio-Render.com.

**Table 1:** Summary of PD animal models.

**Model**	**Characteristics**	**Most Common Induction Protocols and Time Perspective (Acute/Chronic)**	**Main Known Affected Mechanisms**	**Behavioural/** **Symptomatic Changes**	**When to Use this Model**
6-OHDA	Highly oxidisable DA analogue enters the cell through DAT, which allows selective damage to catecholaminergic neurons by cytotoxicity.	Single stereotaxic administration – % of neuronal death is dose-dependent MFB, SN or striatum (Bilateral or unilateral) **Characteristics:** MFB and SN - rapid, generalised degeneration of the damaged nucleus Striatum - progressive loss of dopaminergic neurons of the SNpc Reserpine, an inhibitor of VMAT, can also be injected to protect noradrenergic terminals from 6-OHDA toxicity.	1) intra- or extracellular auto-oxidation of 6-OHDA, which favours the production of hydrogen peroxide, superoxide and hydroxyl radicals; 2) formation of hydrogen peroxide due to the action of monoamine oxidase, and 3) direct inhibition of mitochondrial respiratory chain complex I and IV. Oxidative stress, neurodegeneration, neuroinflammation, and neuronal death by apoptosis. Does not promote α-syn accumulation.	Bradykinesia, cognitive deficits, Enteric Nervous System (ENS) dysfunctions, changes in circadian timing (day/night activity), depressive-like behaviour, changes in gait parameters [112], and nociceptive threshold [22, 113, 114] **Unilateral induction** Forepaw asymmetry use (cylinder test) [23], Rotational motor behaviour (apomorphine [93] or amphetamine tests) (Postural asymmetry).	Evaluate the molecular basis of cytotoxicity and cell processes activated by oxidative stress (local neuroinflammation and DA/catecholaminergic neuron death). Trialling of symptomatic therapies Studies of levodopa-induced dyskinesia and other side effects of dopaminergic drugs Studies of motor and non-motor symptoms.
MPTP	Crosses the BBB. In the brain, astrocytes convert MPTP to a toxic metabolite (MPP^+^) by the enzyme monoamine oxidase-B.	s.c., i.p, i.m., i.v. i.n. and brain injection (SN) Acute - single injection (more common for brain injection) Chronic - multiple injections % of neuronal death is dose/ frequency dependent **Characteristics:** damage to the nigrostriatal pathway, loss of striatal GABAergic neurons and neurons in the VTA and retrorubral nucleus, as well as reactive gliosis.	ATP deprivation, inhibition of mitochondrial complex I and IV, and consequently oxidative stress, activation of the mitochondrial apoptotic cascade, excitotoxicity, inflammation (microglial activation), dysregulation of the UPS, and the formation of inclusion bodies α-syn (mRNA increase and aggregation in the brain) [86] and tau accumulation [89].	Reduced locomotion and rearing (open field) – rodent models - Primate models have similarities to human symptoms.	Induction of bilateral dopaminergic cell loss Closer to human parkinsonism symptoms, including dyskinesia after levodopa therapy (mainly in primates) Testing of potential symptomatic therapies and stem cell therapies.
Rotenone	Crosses the BBB. Isolated from tropical plants, acts as a herbicide and insecticide.	s.c., i.p., and brain injection (SN or striatum – less common) 1.0-2.5 mg/kg Chronic – multiple injections % of neuronal death is dose/ frequency-dependent.	Mitochondrial dysfunction (mitochondrial complex-I inhibitor), oxidative stress. Alteration in lipid and glutathione metabolism (complex I inhibition) α-syn increase, presence of Lewy body-like inclusions TH-positive neuron decrease.	Increased number of falls on the rotarod, increased immobility and decreased climbing on forced swimming test.	Induction of bilateral dopaminergic cell loss. Non-selective for dopaminergic system. Trialling of symptomatic therapies.
Lipopolysaccharide (LPS)	Crosses the BBB. An endotoxin from the outer membrane of bacteria is known as a potent trigger of inflammation.	i.p., i.v. (0.02 mg/kg to 3 mg/kg) or brain injection (2 mg - 10 mg). Acute - single injection Chronic - multiple injections **Characteristics:** Astrocyte and microglia activation, as well as cyclooxygenase-2 (COX-2), inducible nitric oxide synthase (iNOS) and pro-inflammatory cytokine expression.	A robust activation of microglia and astrocytes; release of neurotoxic factors.	Increased number of falls on the rotarod.	Investigation of general neuroinflammatory processes. Induction of bilateral dopaminergic cell loss. Non-selective for the dopaminergic system. Useful in trialling novel PET ligands of neuroinflammation.
Proteasome inhibition	Inhibitors of proteasome activity.	Stereotaxic striato-nigral or i.c.v injections of lactacystin.	Inhibition of proteasome, which leads to α-syn aggregation and DA neuron loss. I.c.v. model also targets noradrenaline and serotonin neurotransmission and leads to neuroinflammation.	Motor dysfunction on catalepsy test, cylinder test and rotational behaviour.	Study deficiencies in proteasome activity, study dopaminergic as well as non-dopaminergic aspects, as well as motor and non-motor symptoms.
Genetic models	Focus on genes with mutations observed in familial PD. Most common target genes: PARK1, LRRK2, PINK1, PARKIN, DJ-1 (PARK7), GBA.	Models based on overexpression or depletion of genes.	Altered mitophagy, dysfunction of the ubiquitin-proteasome pathway, fragmented mitochondria and altered ROS, promoting DA neuron loss.	Increased number of falls on rotarod and decreased overall motor activity in beam walk test [115].	Investigate genes and mutations related to PD development.
Recombinant adeno-associated viral vectors (AAV)	Focus on targeting the SN and promoting an overexpression of α-syn.	Local stereotaxic injection of rAAV in the nigral system and the disease severity can be controlled by adjustment of α-syn dose/expression.	Aggregation of phosphorylated α-syn in terminals, which leads to progressive dopaminergic neuron loss.	Impairment of motor function in the cylinder test, the rotarod test and the open field test.	Useful to understand α-syn toxicity but not prion-like behaviour.
Preformed fibrils (PFF)	Focus on targeting the dopaminergic system and promoting PFF spreading.	Stereotaxic injection of α-syn PFFs or brain lysate/ homogenate from PD patients in the dopaminergic system PFF needs to be properly sonicated to an average of 50nm or smaller before brain injection.	Development of Lewy body-like inclusions promoting DA neuron loss. Misfolded α-syn spreads gradually after injection into areas anatomically connected to striatum and becomes bilaterally located with time. α-syn fibrils promote progressive pathological synaptic impairment prior to neurodegeneration, accompanied by neuroinflammation.	No clear changes in motor behaviour have been reported. Due to the slow development of this model, motor impairments may only become apparent at later timepoints, or with higher amounts of administered PFFs.	Study prion-like behaviour of α-syn propagation Studies of prodromal PD, disease progression, and longitudinal changes Trialling of neuroprotective therapies Useful in trialling novel α-syn PET ligands.

**Table 2:** Radioligands used with the 6-OHDA PD model in the last 5 years.

**Species Age**	**Model Induction Protocol**	**PET Timepoints**	**PET Tracer**
Male C57/BL6J and TREM1-knockout mice 8-12 weeks old [116]	N/A Striatum (unilateral)	7 and 14 days post PD model induction	[^18^F]GE-180, [^64^Cu]TREM1-mAb, [^64^Cu]Isotype control-mAb
Male Long-Evans rats 3 months old [117]	21 μg / 3 μl MFB (unilateral)	1-7 months (every month) - tryptophan metabolism 4 days and 4 weeks - TSPO 1 and 4 weeks - dopaminergic system	7-[^18^F]fluorotryptophan ([^18^F]FTrp), [^18^F]FDOPA, [^18^F]DAA1106
Female Long-Evans WT rats 10-11 weeks old [118]	2x 6 μg / 3 μl MFB and SN (unilateral)	Day 21 - glucose metabolism Day 23 - SV2A	[^18^F]FDG, [^11^C]UCB-J
Male Wistar rats 9 weeks old [119]	21 μg / 5 μL Striatum (unilateral)	2 days after 6-OHDA and after LPS injection (same day)	[^18^F]FEPPA
Male Sprague-Dawley rats N/S [120]	4 μg / 2 μl (SN) and 10 μg / 2.5 μl (MFB)	10 weeks	[^18^F]FDOPA and [^18^F]FMT
Male Sprague-Dawley rats 1 year old [121]	8 μg or 16 μg / 4 μL (partial lesion and full lesion, respectively) MFB (unilateral)	Day 28	[^18^F]FP-(+)-DTBZ
C57Bl6 mice [122]	10 μg / 2 μl Striatum (unilateral)	2 weeks	[^18^F]IAM6067 - sigma 1 receptor - calcium signal modulator
Female Sprague-Dawley rats [96]	2 x 20µg / 4µL Striatum (unilateral)	9 weeks	[^11^C]UCB-J
Male Wistar rats adult [123]	3x – 4 µg / 2 µL Striatum (unilateral)	Day 14	[^18^F]LBT-999
Male Sprague-Dawley rats 8 weeks old [124]	20 μg / 4 μl Striatum (unilateral)	Days 7, 14 and 21	[^18^F]FTPQ
Female Wistar rats 8 weeks old [125]	24 μg / 4 μl Striatum (unilateral)	Day 4, 7, 14, 21, and 28	[^11^C]JNJ-717 and [^18^F]DPA-714#
Male Sprague Dawley rats 6 weeks old [126]	6 µg / 2.3 µl SN (bilateral)	3 weeks	[^3^H]SCH23390 - D1R, [^125^I]Iodosulpride - D2R and [^125^I]7OH-PIPAT - D3R#
C57/BL6J mice 11-15 weeks old [98]	10 μg / 2 μl SN (unilateral)	Days 7, 14 and 21	[^18^F]DPA-714
Male Long-Evans rats 3 months old [127]	21 μg / 3 μl MFB (unilateral)	Day 13-24 and Day 26-29	[^18^F]FDG and [^18^F]FDOPA

**Table 3:** Therapies evaluated by PET and/or autoradiography with the 6-OHDA PD model in the last 5 years.

**Species Age**	**Model Inductions Protocol**	**PET Timepoints**	**PET Tracer**	**Therapeutic Intervention**
Male Hannover-Wistar rats 3 months old [23]	2 x 3 μg / 0.5 μl Striatum (unilateral)	Baseline, days 10 and 30	[^11^C]PBR28 and [^18^F]FDOPA	Motorized running wheel for three days per week - 4 weeks - 10 m/min for 40 minutes - started 2 days after PD induction.
Male Hannover-Wistar rats 2 months old [22]	2 x 9 μg / 1.5 μl Striatum (unilateral)	Day 49	[^3^H]UCB-J, [^3^H]raclopride, [^3^H]DAMGO and [^3^H]PK11195	Treadmill exercise for three days per week - 5 weeks - 10 m/min for 40 minutes - started 15 days after PD induction.
Male Long-Evans rats 3 months old [128]	21 μg / 3 μl MFB (unilateral)	Day 13-24 (glucose metabolism) and Day 26-29 (dopaminergic system)	[^18^F]FDG (OFF and ON) and [^18^F]FDOPA (OFF)	STN-DBS - 55 min of ON condition - monophasic rectangular 60 µs pulses at 130 Hz. The amplitude was initially set to 30 µA and then slowly increased to 50 µA in 5 µA steps.
FemaleWistar rats 8 weeks old [129]	24 μg / 4 μl SN (unilateral)	Baseline and day 16	[^18^F]FPEB	Levodopa-induced dyskinesia - levodopa therapy for 15 days (21 days after PD induction) - 6 mg/kg, i.p., levodopa methyl ester combined with a peripheral DOPA decarboxylase inhibitor, benserazide (12 mg/kg, i.p., benserazide HCl, Sigma) twice daily for 2 weeks.
Male Sprague Dawley rats 442 ± 52 g [130]	12 μg / 2 μl MFB (unilateral)	Baseline and day 22	[^11^C]DASB	Levodopa-induced dyskinesia - levodopa therapy for 21 days (28 days after PD induction) - once daily with 12 mg/kg levodopa and 15 mg/kg benserazide hydrochloride.
Female Wistar rats 8 weeks old [131]	24 μg / 4 μl SN (unilateral)	Days 21-22	[^11^C]preladenant and [^11^C]raclopride	Levodopa-induced dyskinesia - levodopa therapy for 21 days (21 days after PD induction); Levodopa (6 mg/kg) and benserazide-HCl (6 mg/kg) were given twice a day for 15 days.
Male Sprague Dawley rats 8 weeks old [132]	20 μg / 4 μl MFB (unilateral)	Days 1, 15 and 29 (DAT), and day 22 (SERT)	[^18^F]FE-PE2I and 4-[^18^F]ADAM	Dextromethorphan (20 mg/kg) - intraperitoneally twice daily from 7 days before 6-OHDA injection to 28 days after the appearance of a lesion.
Male Wistar rats 285-305 g [133]	24 μg / 4 μl MFB (unilateral)	3 and 6 months after treatment	[^18^F]fallypride	Botulinum neurotoxin A - Intrastriatal injection of botulinum neurotoxin A (BoNT-A) five to six weeks later PD induction.
Male Sprague-Dawley rats 56 days of age [134]	4x 8 μg / 2 μl Striatum (unilateral)	After treatment and	[^18^F]DPA-174, [^18^F]FP-CIT and [^18^F]FPEB	PLX3397, a CSF-1R inhibitor, rapidly inhibits microglial proliferation - daily at 30 mg/kg by oral gavage - from 7 days to 28 days after PD induction.
Female adult Sprague-Dawley rats 8 weeks old [135]	N/A MFB (unilateral)	6 weeks after cell transplantation (10 weeks after PD induction)	[^18^F]FP-CIT	Brain injection of mesenchymal stem cells (MSCs) derived from human placenta MSCs (hpMSCs) or hpMSC-derived neural phenotype cells (hpNPCs) (2×1.5×10^5^/rat).
Male Sprague-Dawley rats 8 weeks old [136]	20 μg / 4 μl MFB (unilateral)	Baseline, 2 weeks after PD induction, and 4 weeks after cell transplantation	[^18^F]FDOPA and [^18^F]FE-PE2I	Intrastriatal transplant of fetal ventral mesencephalic (VM) tissue from rats or pigs (rVM or pVM), with/without a co-graft of Sertoli cells (Cs) (rVM+SCs or pVM+SCs) – 2 weeks after PD induction.
Female NIH nude rats Young [137]	20 μg / 4 μl MFB (unilateral)	Baseline (4 weeks after PD induction), 1month, 3months and 6months post-transplantation	[^18^F]FBCTT, [^18^F]fallypride and [^18^F]FLT	Transplantation of human embryonic stem cell-derived midbrain dopaminergic neurons (hESC-mDAs) - 4×10^5^ cells in 4μl - 1 month after PD induction.
Male Sprague-Dawley rats 8 weeks old [138]	20 μg / 4 μl MFB (unilateral)	Baseline (2 weeks after PD induction) and 4 weeks post-transplantation	4-[^18^F]ADAM	Embryonic day 27 (E27) porcine mesencephalic tissue (~2.5 × 10^5^ cells) - intrastriatal transplantation.
Male Sprague-Dawley rats 8 weeks old [139]	20 μg / 4 μl MFB (unilateral)	Baseline (3 weeks after PD induction) and 8-9 weeks post-transplantation	4-[^18^F]ADAM	Kainic acid bridging and co-graft of rat olfactory ensheathing cells (OECs) and rat E14 embryonic ventral mesencephalic (VM) tissue transplantation into the ipsilateral brain 3 weeks after PD induction.

**Table 4:** Radioligands used with the MPTP PD model in the last 5 years.

**Species Age**	**Model Induction Protocol**	**PET Timepoints**	**PET Tracer**
Male cynomolgus macaques (*Macaca fascicularis*) 3-5 years old (2.5-7.5 kg) [140]	0.5 mg/kg i.v. every two weeks	Baseline and 1 month after reaching a stable motor status	[^11^C]DTBZ and [^18^F]FDG
TREM2-/- mice and C57BL/6 mice 8-12 weeks old [141]	4 i.p. injections at 2- hour intervals (20 mg/kg)	C57BL/6 Days 1, 2, and 7 TREM2-/- mice Day 7	[^11^C]PK1119 and [^11^C]FECIT
Female Göttingen minipigs 1-year-old (25 kg) [51]	3x/week - 4-week, s.c. (18 mg/kg in total - 1 mg/kg for the first 2 weeks and 2 mg/kg for the last 2 weeks)	Baseline, 1, 3, 10 and 14 months post-model induction	[^18^F]FDOPA and [^11^C]DTBZ
Female cynomolgus macaques (*Macaca fascicularis*) 6.3 ± 1.3 years old (2.5-2.8 kg) [142]	Low-dose (0.2 mg/kg), i.m. - continuously until global activity was lower than 8% of baseline data	Baseline, 8, 16, 24, 32, 40 and 48 weeks after model induction	[^18^F]FP-CIT
Male Sunda pig-tailed macaques (*Macaca nemestrina*) 5.4 ± 1.0 years old [143]	0 to 0.31 mg/kg was infused no faster than 1 ml per minute into the right internal carotid artery under angiographic control	Baseline and after 2 months (interval unclear)	[^18^F]FDOPA, [^11^C]DTBZ and [^11^C]CFT
Male rhesus macaques (*Macaca mulatta*) 7.1-9.4 years old (4-6 kg) [144]	0.2 and 0.4 mg/kg (total dose: 8 and 14 mg/kg), i.v., once/week over 4 months until stable Parkinsonian syndrome was observed. The total doses of MPTP administered were between 8 and 14 mg/kg	PET measurements were started at least 2 months after the last treatment with MPTP.	[^11^C]DASB [^18^F]MPPF, [^11^C]PE2I, [^11^C]6MemTyr, [^11^C]raclopride or [^18^F]BCPP-EF (Mitochondrial Complex I Activity)
Rhesus macaques *(Macaca mulatta*) - 3 females and 2 males 5-7 years old [145]	Weekly intramuscular doses (0.2-0.8 mg/kg) starting at 0.2 mg/kg for 18 weeks and increasing to 0.8 mg/kg until stable parkinsonian motor symptoms were observed	Baseline, 8, 16, 24 and 38 weeks after the initiation of MPTP administration.	[^18^F]FEPPA and [^18^F]FECNT
Male rhesus macaques (*Macaca * *mulatta*) 9-13years old (8-19kg) [146]	Unilateral (right) intracarotid artery injection of 3 mg of MPTP-HCl in 20 ml of saline (rate: 1.33 ml/min)	24 months after brain surgery	[^18^F]FEPPA – focusing on CD68-positive microglial/macrophage activation

**Table 5:** Therapies evaluated by PET and/or autoradiography with the MPTP PD model in the last 5 years.

**Species Age**	**Model Induction Protocol**	**PET Timepoints**	**PET Tracer**	**Therapeutic Intervention**
Cynomolgus macaques (*Macaca fascicularis*) 8-14 years old (3.0-4.8 kg) [147]	Once-daily subcutaneous injection of MPTP (0.2 mg/kg)	N/A	[^11^C]raclopride	Pridopidine - 3 doses (15, 20, and 30 mg/ kg)
Cynomolgus macaques (*Macaca fascicularis*) Male (chronic low dose) and female (advanced PD) 11.8 ± 0.8 years old (5.2 ± 0.6 kg) [148]	Early-stage: low doses of MPTP injections (dose range: 0.05-0.25 mg/kg; intravenously) two to three times per week for up to 15 months. Advanced-stage: continuous infusion of MPTP using subcutaneous osmotic mini-pumps (0.5 mg/24 hours) for around 6 months	N/A	[^11^C]-PXT012253	Foliglurax - PXT002331 (in water) was administered at dose levels of 2 and 25 mg/kg as a single dose on days 1 and 8 and twice daily on days 2 through 7
Male C57BL/6 mice 5-8 weeks old (25-35 g) [149]	Intraperitoneally (i.p.) injected with a single daily dose of 25 mg/kg MPTP hydrochloride solution for 5 consecutive days (days 1-5)	Baseline PET one day before MPTP treatment (day 0), a second PET scan one day before magnolol treatment (day 10), and a third PET scan on the day following the final treatment (day 17)	[^18^F]DTBZ	Magnolol (10 mL/kg, i.p.) - single daily dose for 6 days after the final MPTP treatment
Cynomolgus macaques (*Macaca fascicularis*) 4 males and 5 females (3.3-8.0 kg) [150]	Subacute type: MPTP was administered (0.5 mg/kg) for 3 consecutive days and then every 2-3 days while motor signs were monitored (a total of 5-9 days). Subchronic type: MPTP was administered (0.3 mg/kg) for 2 consecutive days and then every 6-8 days while motor signs were monitored (a total of 30-70 days)	N/A	[^11^C]PE2I	Lentiviral vector therapy (Calbindin) - 1 to 2 months before MPTP was systemically administered
Rhesus macaques (*Macaca mulatta*) 10-14 years old (8-19 kg) [151]	Unilateral (right) intracarotid artery (ICA) injection of 3-4 mg of neurotoxin MPTP-HCl	12–18 months post-cell transplantation	[^11^C]DTBZ	Cell therapy: received induced pluripotent stem cell DA in the basal ganglia ipsilateral to the MPTP ICA injection (right)- 1-3 years after MPTP model induction
Rhesus macaques (*Macaca mulatta*) - 14 males and 2 females 8-22 years old (9.1±1.7 kg for PD and 9.7±2.7 kg) [110]	Chronic intravenous administration of MPTP over several months	The parkinsonian animals were evaluated before and 6-48 months after unilateral striatal implantation	[^18^F]FDG	Human retinal pigment epithelial cells - hRPE - hRPE-GM or sham (GM only) were implanted unilaterally in the striatum in the fully recovered and stable animals (*i.e*., motor scores unchanged for 3-4 months)
C57BL/6 mice 8 weeks old [152]	N/A	N/A	[^89^Zr]hNSCs	Human neural stem cells (hNSCs) - nasal and striatal administration
Male Rhesus macaques (*Macaca mulatta*) 9-13 years old (8-19 kg) [146]	Unilateral (right) intracarotid artery injection of MPTP	Twenty-four months of follow-up	[^18^F]FEPPA	Induced pluripotent stem cell - 3 to 12 months later, the monkeys received injections of allogeneic iPSC-mDA
Male Cynomolgus macaques (*Macaca fascicularis*) 2-3-years-old [153]	The animals were injected intravenously with MPTP hydrochloride (0.4 mg kg−1 as a free base; Sigma-Aldrich) twice a week until they observed persistent Parkinsonian symptoms	Before, 2 weeks and 1, 3, 6, 12, 18, and 24 months after cell transplantation	[^11^C]PK11195, [^11^C]KTP-Me, [^18^F]FLT	Induced pluripotent stem cell
Cynomolgus macaques (*Macaca fascicularis*) (2.5-3.5 kg) [154]	Intravenous injections of MPTP HCl (0.4 mg/kg) twice a week until persistent Parkinsonian behavioral symptoms became evident	N/A	[^18^F]DOPA	Induced pluripotent stem cell
C57bl/6J mice 8 weeks old (22.4 ± 0.8 g) [155]	MPTP (15 mg/kg; Sigma- Aldrich, St. Louis, MO, USA) intraperitoneally for 5 consecutive days	Day 0 (baseline; scan 1), day 6 (scan 2), and day 13 (scan 3)	[^18^F]FE-PE2I	Fas-associated factor 1 inhibitor - KM-819. Oral administration (20 mg/kg) for 6 consecutive days, starting from 48 h after the last dose of MPTP
Male cynomolgus macaques (*Macaca fascicularis*) 2.5 ± 0.1 years old; 3.48 kg ± 0.1 kg) [156]	Intramuscular injections of 0.25 mg/kg of MPTP for 7 consecutive days, as previously described	Baseline, post-MPTP lesioning, and at 6 months post-vector administration	6-[^18^F]-fluoro-L-m-tyrosine	Gene therapy - OXB-102 (a lentiviral vector with an optimized expression cassette for DA biosynthesis)
Cynomolgus macaques (*Macaca fascicularis*) 3-5 years old (4-6 kg) [157]	Intramuscular MPTP injections (mean total dose of 1.55 mg/kg)	1) Baseline, 2) after MPTP and just before the first levodopa period (post-MPTP), 3) after the first period of levodopa treatment (post-levodopa1), 4) after a two-month washout period and before MDMA (pre-MDMA), 5) after MDMA and just before the second period of levodopa exposure (post-MDMA) and 6) after the second levodopa period (post-levodopa2)	[^11^C]PE2I and [^11^C]DASB	Levodopa-inducing dyskinesia – 2 months after MPTP intoxication, all monkeys received intra-muscular injections of L-3,4-levodopa twice daily for 2 months
Rhesus macaques (*Macaca mulatta*) - 3 females and 2 males 5-7 years old [145]	Weekly doses of intramuscular MPTP (0.2-0.8 mg/kg, Sigma-Aldrich) starting at 0.2 mg/kg for 18 weeks and increasing to 0.8 mg/kg to maintain a parkinsonian state	Baseline and at approximately 8 (PET I), 16 (PET II), 24 (PET III) and 38 weeks (PET IV) after the initiation of MPTP administration	[^18^F]FEPPA	Tumor necrosis factor (TNF) inhibitor (XPro1595) - At 11 weeks, subcutaneous treatment of XPro1595 (10 mg/kg) started and continued every 3 days

**Abbreviation:** N/A – not available.

## LIST OF ABBREVIATIONS

A2AR: Adenosine A2A Receptor
AAAD: Aromatic L-amino Acid Decarboxylase
AAV: Recombinant Adeno-Associated Virus
ALP: Autophagy-Lysosome Pathway
ATP: Adenosine Triphosphate
BAC: Bacterial Artificial Chromosome
BBB: Blood Brain Barrier
CB2: Cannabinoid Receptor Type 2
CNS: Central Nervous System
COX-2: Cyclooxygenase-2
CSF1R: Colony-stimulating Factor 1 Receptor
DA: Dopamine
DAT: Dopamine Transporter
DBS: Deep Brain Stimulation
DDC: Levodopa Decarboxylase
DMV: Motor Nucleus of the Vagus Nerve
GBA: Glucosylceramidase Beta 1
GCase: Glucocerebrosidase
GLUT: Glucose Transporter
GWAS: Genome-Wide Association Study
Iba-1: Ionized Calcium-binding Adapter Molecule 1
ICV: Intracerebroventricular
iNOS: Inducible Nitric Oxide Synthase
KO: Knockout
LPS: Lipopolysaccharides
LRRK2: Leucine-Rich Repeat Kinase 2
MAO-B: Monoamine Oxidase B
MBCTT: (1R,2S,3S,5S)-8-Azabicyclo[3.2.1]octane-2-carboxylic acid, 3-(4-methylphenyl)-8-[4-[(methylsulfonyl)oxy]-2-butyn-1-yl]-methyl ester
MFB: Median Forebrain Bundle
MPP^+^: 1-Methyl-4-phenylpyridiniu
MPTP: 1-Methyl-4-phenyl-1,2,3,6-tetrahydropyridine
MRI: Magnetic Resonance Imaging
NHP: Non-Human Primate
6-OHDA: 6-Hydroxydopamine
P2X7: P2X Purinoceptor 7
PD: Parkinson’s Disease
PET: Positron Emission Tomography
PFF: Pre-Formed Fibril
PINK1: Pten-Induced Kinase 1
PRKN or PARK2: Parkin
PSI: Proteasome Inhibitor
RBD: REM Sleep Behavior Disorder
ROS: Reactive Oxygen Species
S1P: Sphingosine-1-Phosphate
SN: Substantia Nigra
SNP: Sodium Nitroprusside
SNpc: Substantia Nigra *pars compacta*
SPECT: Single-Photon Emission Computerized Tomography
STN: Subthalamic Nuclei
SV2A: Synaptic Vesicle Glycoprotein 2A
α-syn: Alpha-Synuclein
TH: Tyrosine Hydroxylase
TSPO: Translocator Protein 18 KDa
UPS: Ubiquitin-Proteasome System
VMAT2: Vesicular Monoamine Transporter 2
VMAT: Vesicular Monoamine Transporters
VTA: Ventral Tegmental Area

## LIST OF RADIOLIGANDS

**Neuroinflammation**

**Translocator Protein 18kDa (TSPO)**

[^11^C]PBR28 - N-Acetyl-N-(2-[^11^C]Methoxybenzyl)-2-Phenoxy-5-Pyridinamine

[^11^C]PK11195 - 1-(2-Chlorophenyl)-N-methyl-N-(1-methylpropyl)-3-isoquinolinecarboxamide C-11

[^18^F]DPA-714 - N,N-diethyl-2-[2-[4-(2-(^18^F)fluoranylethoxy)phenyl]-5,7-dimethylpyrazolo [1,5-a]pyrimidin-3-yl]acetamide

[^18^F]DPA-714 - N,N-diethyl-2-[2-[4-(2-(^18^F)fluoranylethoxy) phenyl]-5,7-dimethylpyrazolo [1,5-a]pyrimidin-3-yl]acetamide

[^18^F]DAA1106 - (N-(2,5-dimethoxybenzyl)-N-(5-fluoro-2-phenoxyphenyl)acetamide)

[^18^F]FEPPA - N-[[2-(2-(^18^F)fluoranylethoxy)phenyl]methyl]-N-(4-phenoxypyridin-3-yl)acetamide

[^18^F]FTPQ - [^18^F]-2-(4-fluoro-2-(p-tolyloxy)phenyl)-1,2-dihydroisoquinolin-3(4H)-one

[^18^F]GE-180 - (4S)-N,N-diethyl-9-(2-(^18^F)fluoranylethyl)-5-methoxy-1,2,3,4-tetrahydrocarbazole-4-carboxamide

**Colony-Stimulating Factor 1 Receptor**

[^11^C]CPPC - 5-cyano-N-(4-(4-[^11^C]Methylpiperazin-1-yl)-2-(Piperidin-1-yl)Phenyl)Furan-2-carboxamide [^11^C]GW2580 - 5-[[3-methoxy-4-[(4-methoxyphenyl)methoxy]phenyl]methyl] pyrimidine-2,4-diamine C11

**P2X7 Receptor**

[^11^C]JNJ-717 - [^18^F]JNJ-64413739

**Cyclooxygenase (COX)**

[^11^C]KTP-Me - [^11^C]ketoprofen methyl ester

**Tryptophan Metabolism**

[^18^F]FTrp - 7-[^18^F]fluorotryptophan

**Dopaminergic System**

**Dopamine synthesis**

[^18^F]FDOPA - (2S)-2-amino-3-(2-(^18^F)fluoranyl-4,5-dihydroxyphenyl)propanoic acid

6-[^18^F]-fluoro-L-m-tyrosine - (2S)-2-amino-3-(2-(^18^F)-fluoranyl-5-hydroxyphenyl)propanoic acid L-[β-^11^C]DOPA

**Dopamine Receptors**

[^11^C]raclopride - 3,5-dichloro-N-[[(2S)-1-ethylpyrrolidin-2-yl]methyl]-2-hydroxy-6-(1^11^C)methoxybenzamide - D2R

[^11^C]SCH23390 - (5R)-8-chloro-3-(1^11^C)methyl-5-phenyl-1,2,4,5-tetrahydro-3-benzazepin-7-ol - D1R

[^125^I]7OH-PIPAT - 2-[(2E)-3-iodoprop-2-en-1-yl](propyl) aminotetralin-7-ol - D3R

[^125^I]Iodosulpride - N-[[1-(cyclopropylmethyl)pyrrolidin-2-yl]methyl]-5-ethylsulfonyl-4-iodo-2-methoxybenzamide - D2R

[^18^F]fallypride - 5-(3-(^18^F)fluoranylpropyl)-2,3-dimethoxy-N-[[(2S)-1-prop-2-enylpyrrolidin-2-yl]methyl]benzamide - D2/3R

[^3^H]-SCH23390 - 8-chloro-3-methyl-5-phenyl-1,2,4,5-tetrahydro-3-benzazepin-7-ol - D1R

[^3^H]raclopride - 3,5-dichloro-N-[[(2S)-1-ethylpyrrolidin-2-yl]methyl]-2-hydroxy-6-methoxybenzamide - D2R

**Dopamine Transporter**

[^11^C]DTBZ - 2S,3S,11bS)-10-methoxy-9-(1^11^C)methoxy-11b-methyl-3-(2-methylpropyl)-1,2,3,4,6,7-hexahydrobenzo [a]quinolizin-2-ol

[^11^C]FECIT - [^11^C]N-2-fluoroethyl-2-β-carbomethoxy-3-β-(4-iodophenyl)-nortropane

[^11^C]PE2I - methyl (1R,2S,3S,5S)-8-[(E)-3-iodoprop-2-enyl]-3-(4-methylphenyl)-8-azabicyclo[3.2.1]octane-2-carboxylate C11

[^11^C]β-CFT - 2-β-carbomethoxy-3-β-(4-fluorophenyl) tropane 11C

[^18^F]-FP-CIT - methyl (1R,2S,3S,5S)-8-(3-(^18^F)fluoranyl-propyl)-3-(4-iodophenyl)-8-azabicyclo[3.2.1]octane-2-carboxylate

[^18^F]DTBZ - (2R,3R,11bR)-9-(3-(18F)fluoranylpropoxy)-10-methoxy-3-(2-methylpropyl)-2,3,4,6,7,11b-hexahydro-1H-benzo[a]quinolizin-2-ol

[^18^F]FBCTT - nucleophilic substitution of the MBCTT mesylate precursor with [^18^F]fluoride

[^18^F]FE-PE2I - 2-fluoroethyl (1S,2S,3S,5S)-8-[(E)-3-iodoprop-2-enyl]-3-(4-methylphenyl)-8-azabicyclo[3.2.1] octane-2-carboxylate - dopamine transporter

[^18^F]FMT - (2S)-2-amino-3-(2-(^18^F)fluoranyl-5-hydroxy-phenyl)propanoic acid [^18^F]LBT-999 - methyl (1R,2S,3S,5S)-8-[(E)-4-fluorobut-2-enyl]-3-(4-methylphenyl)-8-azabicyclo [3.2.1]octane-2-carboxylate - Dopamine transporter

[^18^F]FECNT - ^18^F-labeled 2beta-carbomethoxy-3beta-(4-chlorophenyl)-8-(2-fluoroethyl)

**Glucose Metabolism**

[^18^F]FDG - (2S,3R,4S,5S,6R)-3-(^18^F)fluoranyl-6-(hydroxymethyl)oxane-2,4,5-triol

**Synaptic Density**

[^11^C]UCB-J - (R)-1-((3-((11)C-methyl-^11^C)pyridin-4-yl)methyl)-4-(3,4,5-trifluorophenyl)pyrrolidin-2-one

**Opioidergic System**

[^3^H]DAMGO - (2S)-2-amino-N-[(2R)-1-[[2-[[(2S)-1-(2-hydroxyethylamino)-1-oxo-3-phenylpropan-2-yl]-methylamino]-2-oxoethyl]amino]-1-oxopropan-2-yl]-3-(4-hydroxyphenyl)propanamide

**Glutamatergic System**

[^18^F]FPEB - 3-(^18^F)fluoranyl-5-(2-pyridin-2-ylethynyl) benzonitrile

[^11^C]-PXT012253 - N-(4-chloro-3-(1^11^C)methylsulfanyl-phenyl)pyridine-2-carboxamide

**Serotoninergic System**

[^11^C]DASB - 3-amino-4-[2 [methyl((1^11^C)methyl) amino] methyl]phenyl]sulfanylbenzonitrile

4-[^18^F]-ADAM - N,N-Dimethyl-2-(2-Amino-4-(^18^F)Fluorophenylthio)Benzylamine

[^18^F]MPPF - 4-(fluoro-^18^F)-N-(2-(4-(2-methoxyphenyl) piperazin-1-yl)ethyl)-N-(pyridin-2-yl)benzamide

**Adenosine Receptor**

[^11^C]preladenant - 2-(2-Furanyl)-7-[2-[4-[4-(2-methoxy-ethoxy)phenyl]-1-piperazinyl]ethyl]7H-pyrazolo [4,3-e][1,2,4]triazolo [1,5-c]pyrimidine-5-amine

[^18^F]FLUDA - a deuterated isotopologue of [^18^F]FESCH [^18^F]FESCH - ^18^F-labeled pyrazolo [4,3-e]-1,2,4-triazolo [1, 5-c]pyrimidine

**Cell Therapy**

[^64^Cu]TREM1-mAb - antibody against receptor expressed on myeloid cells 1 (TREM1)

[^89^Zr]hNSCs - Human Neural Stem Cells

**Cell-Proliferation (Tumor-Specific)**

[^18^F]FLT - 1-[(2R,4S,5R)-4-(^18^F)fluoranyl-5-(hydroxymethyl) oxolan-2-yl]-5-methylpyrimidine-2,4-dione

**ATP-Gated Ion Channel**

[^3^H]JNJ- 54232334

**Receptor Expressed on Myeloid Cells 1**

[^64^Cu]Isotype control-mAb - Isotype control monoclonal antibody

**Bone Marrow-Derived Macrophages**

[^64^Cu]BMM - bone marrow-derived macrophages

**Calcium Signal Modulator**

[^18^F]IAM6067 (sigma 1 receptor)

**Alpha-Synuclein**

[^11^C]MODAG-001

[^18^F]2FBox

[^18^F]ACI-12589

**Reactive Oxygen Species**

[^11^C]hydromethidine - N-methyl-2,3-diamino-6-phenyl-dihydrophenanthridine ^11^C

**Cannabinoid Receptor Type 2**

[^11^C]A-836339 - N-[3-(2-(1^11^C methoxyethyl)-4,5-dimethyl-1,3-thiazol-2-ylidene]-2,2,3,3-tetramethylcyclo-propane-1-carboxamide
